# Transcriptional dynamics of CD8^+^ T-cell exhaustion in immune checkpoint inhibitor resistance at single-cell resolution

**DOI:** 10.1186/s12943-025-02468-7

**Published:** 2025-12-11

**Authors:** Tzu-Yang Tseng, Ching-Hung Hsieh, Hsuan-Cheng Huang, Yu-Ching Wu, Chiun Hsu, Chia-Lang Hsu, Da-Liang Ou, Hsueh-Fen Juan

**Affiliations:** 1 Department of Life Science, National Taiwan University, No. 1, Sec. 4, Roosevelt Rd., Taipei, 106 Taiwan; 2 Graduate Institute of Oncology, National Taiwan University, No.2, Syujhou Rd., Taipei, 100 Taiwan; 3 Institute of Biomedical Informatics, National Yang Ming Chao Tung University, No. 155, Sec. 2, Linong St., 112 Taipei, Taiwan; 4 Department of Medical Research, National Taiwan University Hospital, No.7, Chung Shan S. Rd., Taipei, 100 Taiwan; 5 Department of Medical Oncology, National Taiwan University Cancer Center, No. 57, Ln. 155, Sec. 3, Keelung Rd., 106 Taipei, Taiwan; 6 Graduate Institute of Medical Genomics and Proteomics, National Taiwan University, No. 1, Sec. 1, Ren’ai Rd., 100 Taipei, Taiwan; 7 Center for Computational and Systems Biology, National Taiwan University, No. 1, Sec. 4, Roosevelt Rd., 106 Taipei, Taiwan; 8 YongLin Institute of Health, National Taiwan University, No. 49, Fanglan Rd., 106 Taipei, Taiwan; 9 Graduate Institute of Biomedical Electronics and Bioinformatics, National Taiwan University, No. 1, Sec. 4, Roosevelt Rd., 106 Taipei, Taiwan; 10 Center for Advanced Computing and Imaging in Biomedicine, National Taiwan University, No. 1, Sec. 4, Roosevelt Rd., 106 Taipei, Taiwan

**Keywords:** Next generation sequencing – NGS, T cell, Immune checkpoint inhibitor, Gene expression profiling – GEP, Hepatocellular carcinoma

## Abstract

**Background:**

Tumor-specific CD8^+^ T lymphocytes play a critical role in anticancer immunity but frequently become dysfunctional and exhausted within the immunosuppressive tumor microenvironment. Although immune checkpoint inhibitors can restore T-cell activity, resistance to these treatments remains a significant challenge. Therefore, understanding the transcriptional and regulatory mechanisms underlying CD8^+^ T-cell exhaustion is crucial for the development of effective therapies.

**Methods:**

We developed two murine models of acquired immune checkpoint inhibitor resistance through prolonged anti-PD1 treatment. To gain insight into CD8^+^ T-cell exhaustion, we performed single-cell multiomics analysis, including both scRNA-seq and scATAC-seq, to capture gene expression profiles and chromatin accessibility. Moreover, we collected three external datasets to validate the results in silico. We further assessed the therapeutic potential of Runx2 through marker expression and cytotoxicity assays.

**Results:**

Our single-cell analysis revealed distinct T-cell subsets, including early and terminally exhausted populations, along with their exhaustion trajectories. Runx2 was identified as a key transcription factor associated with CD8^+^ T-cell exhaustion in both models and correlated with immunotherapy response in clinical data. Additionally, functional marker expression and cytotoxicity assays demonstrated that inhibiting Runx2 improved CD8^+^ T-cell cytotoxicity.

**Conclusions:**

These findings highlight the role of Runx2 as a crucial regulator of CD8^+^ T-cell exhaustion in the context of prolonged immune checkpoint inhibitor treatment. Targeting Runx2 may provide a novel strategy to overcome immune checkpoint inhibitor resistance and enhance therapeutic efficacy, offering promising avenues for combination therapies.

**Supplementary Information:**

The online version contains supplementary material available at 10.1186/s12943-025-02468-7.

## Background

Immune checkpoint inhibitors (ICIs) represent a groundbreaking approach in cancer therapy, harnessing the immune system to target and destroy malignant cells. ICIs, including inhibitors of programmed death-1 (PD-1), programmed death-ligand 1 (PD-L1), and cytotoxic T-lymphocyte-associated antigen 4 (CTLA-4), work by blocking the inhibitory signals that prevent T cells from attacking cancer cells. These therapeutics have been approved for the treatment of various types of cancers [1]. However, the efficacy of ICI therapy is limited, and the detailed mechanisms of resistance to ICIs, especially acquired resistance, are not fully understood.

A significant mechanism associated with resistance to ICI therapy is T-cell exhaustion. T-cell exhaustion is characterized by the progressive loss of T-cell effector functions, sustained expression of inhibitory receptors, and distinct transcriptional and epigenetic profiles [2]. Tumor-infiltrating exhausted CD8^+^ T cells are associated with the response to ICI therapy and are considered potential biomarkers for predicting whether ICI therapy will be effective in an individual patient [3]. Although ICIs reinvigorate the effector functions of exhausted CD8^+^ T cells, prolonged activation of these T cells might lead to acquired resistance [4]. Understanding the mechanisms of T-cell exhaustion and developing strategies to reverse or mitigate this outcome are critical for improving the outcomes of ICI therapy.

In this study, we investigated T-cell infiltration in hepatocellular carcinoma (HCC). HCC is the most common form of primary liver cancer and a global health concern with increasing incidence and mortality [5]. ICI single-agent and combination therapies have been approved as first- and second-line treatments for advanced HCC [6]. However, approximately 20–40% of HCC patients treated with ICI-based regimens exhibit primary resistance, and a substantial number of patients develop acquired resistance during treatment [7]. CD8^+^ T cells play a critical role in the tumor microenvironment of HCC and primary resistance to ICI-based therapy [8, 9]. Therefore, to address acquired resistance to ICIs in HCC, it is necessary to investigate the regulatory network of CD8^+^ T cells in detail.

To elucidate the mechanism of acquired T-cell resistance to ICI therapy, we established two models and conducted single-cell sequencing, including single-cell ATAC sequencing (scATAC-seq) and single-cell RNA sequencing (scRNA-seq), to gain comprehensive insights into the gene regulatory mechanisms underlying the transcriptional regulation process in progressive T-cell exhaustion related to acquired resistance to ICIs. We observed a specific exhausted T-cell population and revealed the path of T-cell exhaustion after the development of acquired resistance. Furthermore, we revealed a crucial gene regulatory network (GRN) through the identification of important transcription factors (TFs) and their downstream target genes that direct CD8^+^ T cells into the progressive exhaustion process. We also analyzed three external datasets and performed additional experiments to confirm the findings. This integrated single-cell analysis revealed that Runx2 is an important TF in the T-cell exhaustion process, a finding that may help improve the effectiveness of HCC therapies and warrants further exploration in the quest for more comprehensive and impactful treatment options.

## Methods

### Model establishment

All the mice were housed in specific pathogen-free areas of the Laboratory Animal Center at the National Taiwan University College of Medicine. All animal experiments received approval from the Institutional Animal Care and Use Committee (IACUC) of the College of Medicine, National Taiwan University (IACUC number: 20210100). We adhered to the guidelines outlined in the Guide for the Care and Use of Laboratory Animals. We obtained C57BL/6 mice from the National Laboratory Animal Center and P14 mice (strain #: 004694) from The Jackson Laboratory.

We constructed two distinct models to elucidate the crucial gene regulatory network that causes T-cell exhaustion. We first designed an in vivo mouse model to examine the differential effects of anti-PD1, an ICI that targets PD1, on T cells. We inoculated C57BL/6 mice subcutaneously with 2 × 10^6^ Hepa1-6 hepatocellular carcinoma cells (ATCC Cat# CRL-1830). Mice were randomly assigned to one of two groups, anti-PD1 treatment or isotype, when tumors grew to an average volume of 200 mm^3^. For the short-term treatment experiment, either an anti-mouse PD1 antibody (clone RMP1–14, Bio X Cell Cat# BE0146) or an isotype control antibody (clone 2A3, Bio X Cell Cat# BE0089, West Lebanon, New Hampshire, USA) was administered once every two days from day 15 to day 19, and the mice were sacrificed on day 20. Conversely, in the long-term treatment experiment, treatments were administered every other day from day 15 to day 19 and then once per week starting from day 20, and the mice were sacrificed on day 57. Tumor samples were collected from each group. Two mice per condition were used for further studies, with tumor samples harvested for single-cell RNA sequencing.

We also designed an ex vivo model to recapitulate different phenotypic subsets of CD8^+^ T cells. We stimulated CD8^+^ T-cell exhaustion from P14 mice, in which CD8^+^ T cells can specifically recognize the gp33 peptide. Different conditions were used to generate four types of T cells from the collected CD8^+^ T cells: naïve, active, exhausted at 72 h, and exhausted at 96 h. For naïve T-cell generation, we cultured T cells for four days with only IL-2. For the active T cells, we treated T cells with a high dose of gp33 peptide (1 µM) once a day after seeding and subsequently cultured them for 24 h with medium to generate the active T-cell group.

Exhausted CD8^+^ T cells were produced by repeated stimulation with the gp33 peptide. We treated T cells with low-dose gp33 peptide (10 nM) once daily and cultured them for three and four days to obtain the exhausted 72 h and 96 h groups.

For intracellular cytokine staining, inducing T cells to suitable conditions, we added PMA (50ng/ml) and ionomycin (500ng/ml) to stimulate T cells and simultaneously treated transport inhibitor (BD) to accumulate cytokine preventing secretion for the final 4 h prior to staining. Then we collected cells to stain specific markers for functional analysis. For single-cell multiome sequencing, CD8^+^ T cells from each group were collected via a cell sorter (BD FACSAria III) and separated for single-cell multiome sequencing, including scRNA-seq and scATAC-seq.

Additionally, we built a Hepa1-6 anti-PD1 resistant model to answer associated questions. We inoculated Hepa1-6 cells into the right flank of C57BL/6 mice. When the average tumor volume reached 200 mm³, we began treatment with either anti-PD1 antibody or isotype control. When tumor occurred resistant for anti-PD1 treatment, we sorted cancer cells by cell sorter (BD FACSAria III) and cultured to expand cancer cells. Using the resistant cell line in vivo study, we implanted the cells into mice and treated anti-PD1, CADD522 (25 mg/kg twice a week, ip), or isotype control to research RUNX2 mechanism.

## Flow cytometry

The collected samples were washed with PBS and then stained with Zombie NIR™ (BioLegend Cat# 423106), a fixable viability dye, for 15–30 min at room temperature. Next, we added an Fc blocker (BioLegend Cat# 101320) for 10 min at 4 °C. The surface markers were subsequently stained at the appropriate concentrations for 30 min at 4 °C. After allotting, the samples were washed with cell staining buffer (BioLegend), and 200 µL of 1% paraformaldehyde (Boster Bio) was added to fix the samples. If intracellular staining was needed, we added 200 µL of permeabilization buffer (eBioscience) to permeabilize the cells overnight at 4 °C, followed by washing with cell staining buffer. On the following day, the cells were washed with permeabilization buffer (eBioscience) and stained with intracellular markers for 30–60 min at 4 °C. The samples were then washed again with permeabilization buffer and resuspended in 200 µL of 1% paraformaldehyde. The results were analyzed by BD LSRFortessa Cell Analyzer and FlowJo software (v10.10).

The following flow-staining antibodies were obtained from BioLegend: CD3-FITC (BioLegend Cat# 155604), CD3-APC (BioLegend Cat# 100236), CD3-PE (BioLegend Cat# 100206), CD8-APC (BioLegend Cat# 100712), CD8-APC-Cy7 (BioLegend Cat# 100714), CD45-BV605 (BioLegend Cat# 103140), IFN-γ-PE (BioLegend Cat# 505808), TNF-α-PE (BioLegend Cat# 506305), TNF-α-Alexa Fluor^®^ 647 (BioLegend Cat# 506314), CD4-BV605 (BioLegend Cat# 100548), CD4-BV510 (BioLegend Cat# 100559), NK1.1-BV421 (BioLegend Cat# 108731), IL-2-PE (BioLegend Cat# 503808), PD-1-BV711 (BioLegend Cat# 135231), LAG3-PerCP/Cyanine5.5 (BioLegend Cat# 125212), EOMES-PE-Cy7 (BioLegend Cat# 157705), and T-bet-PE (BioLegend Cat# 644823). The following flow-staining antibodies were obtained from eBioscience: TOX-PE (Thermo Fisher Scientific Cat# 12-6502-82), Granzyme B-PE (Thermo Fisher Scientific Cat# 12-8898-82), and CD19- eFluor™ 660 (Thermo Fisher Scientific Cat# 50-0193-82). The flow-staining reagents obtained from BD included Annexin V-FITC antibody (BD Biosciences Cat# 556547) and propidium iodide (PI).

### Cytotoxicity assay

We collected CD8^+^ T cells from P14 mice, which specifically recognize the gp33 peptide, and induced them to have different and suitable statuses. During this process, CD8^+^ T cells were treated with either vehicle or CADD522, a RUNX2 inhibitor, and subsequently used as effector cells. Then, we seeded 1 × 10^5^ Hepa1‒6 gp33 cancer cells in 12-well plates to serve as target cells. We cocultured CD8^+^ T cells with Hepa1-6 gp33 cancer cells at a predetermined effector-to-target (E: T) ratio for 30 h. After this period, we harvested and stained the cells with the CD45 marker and a live/dead dye. The cytotoxic capacity was evaluated by flow cytometry, which analyzes the percentage of dead cancer cells by gating on CD45^−^ and Zombie NIR^+^ populations. We calculated the percentage of cytotoxicity via the following formula to normalize the spontaneous death of cancer cells:

$$\:\frac{\mathrm{total}\:\mathrm{dead}\:\mathrm{of}\:\mathrm{cancer}\:\mathrm{cells}\:\left(\%\right)-\mathrm{spontaneous}\:\mathrm{dead}\:\mathrm{of}\:\mathrm{cancer}\:\mathrm{cells}\:\left(\%\right)}{1-\mathrm{spontaneous}\:\mathrm{dead}\:\mathrm{of}\:\mathrm{cancer}\:\mathrm{cells}\:\left(\%\right)}$$

### Single-cell sequencing

For the in vivo model, we conducted scRNA-seq for each sample after cell sorting using BD Biosciences FACSAria III Cell Sorter. To generate single-cell gel beads in emulsion (GEM), cell suspensions were loaded onto 10x Genomics Chromium Controller Genetic Analyzer. ScRNA-seq Libraries were generated following the instructions provided with the Chromium Next GEM Single Cell 3’ Kit v3.1 from 10x Genomics. The barcoded single-cell transcriptome Library pool was sequenced via the Illumina NovaSeq 6000 Sequencing System.

To generate a single-cell multiome dataset from an ex vivo T-cell model, CD8^+^ T cells were subjected to nuclear isolation followed by the use of the Chromium Next GEM Single Cell Multiome ATAC + Gene Expression Kit (10x Genomics) according to the instructions provided by the manufacturer. Sequencing was then conducted on the Illumina Illumina NovaSeq 6000 Sequencing System to generate the single-cell multiome dataset.

### Preprocessing and quality control of scRNA-seq data from a mouse model

CellRanger software was employed for demultiplexing raw reads obtained from the 10x Genomics single-cell RNA-seq platform with default parameters. Single-cell gene count matrices were generated by aligning reads to the mouse reference genome and assigning them to cells on the basis of unique molecular identifier (UMI) barcodes. The preprocessing of count matrices was performed via the Scater R package (v1.22.0). Cells with a median absolute deviation (MAD) 3 times greater than the median were removed. Cells were also removed if the mitochondrial gene count ratio was greater than 5% or if the gene count per cell was lower than 10. The genes expressed in 100 or more cells were retained for further analysis. Concurrently, cells identified as doublets via DoubletFinder (v2.0.3) on the basis of gene expression profiles were removed to mitigate the impact of technical artifacts.

### Normalization, dimensional reduction and cell clustering

The Seurat R package (v4.0.3) was utilized for gene count normalization, dimensional reduction, cell clustering and further analysis [10]. The gene expression of each cell was normalized via the default scale factor of 10,000 and log transformation. For dimensional reduction and subsequent analysis, we initially selected and scaled the expression levels of the top 3,000 highly variable genes (HVGs). Principal component analysis (PCA) was then conducted on these HVGs. To determine a suitable principal component (PC) cutoff for visualization, we selected the PC cutoff on the basis of a cumulative percentage greater than 90% and a variation smaller than 5%. Two-dimensional visualization was achieved via unified manifold approximation and projection (UMAP) of significant PCs [11]. For cell clustering, shared nearest neighbor construction was performed, and clusters were assigned to each cell via the Leiden algorithm implemented in Seurat [12].

### Differential gene expression analysis and T-cell subtype annotation

To characterize the T-cell subtypes within the mouse model, the FindMarkers function was used with the Wilcoxon rank-sum test in Seurat to conduct differential gene expression analysis on the basis of the cell clustering results. Concurrently, we gathered canonical marker gene sets for well-known T-cell subtypes and defined the T-cell subtypes of each cell cluster according to the expression level of each subtype marker.

### Module activity analysis of signature gene sets and cell cycle scoring

To gauge the gene module activity associated with T-cell functions, including immune checkpoint signaling and effector function, we examined the expression levels of selected gene sets. We compiled sets of selective marker genes corresponding to each T-cell function. The Seurat AddModuleScore function, which relies on marker genes obtained from prior studies, was instrumental in assessing the activity of these gene sets [13–15]. The module activity was visually represented via a ridge plot, allowing the elucidation of differences between T-cell subtypes. The cell cycle phase score of the mouse dataset was calculated via the CellCycle scoring function based on canonical markers.

### Cell developmental RNA velocity

RNA velocity analysis was used to calculate the gene-specific rates of transcription. To extract the necessary unspliced and spliced reads for each cell, we utilized binary alignment map (BAM) files from scRNA-seq as inputs for kallisto (v0.46.1) and loompy (v3.0.6) through the command line interface. Kallisto was used to determine transcriptomic expression via the mouse reference genome GRCm38 with annotation from GENCODE (vM23) [16]. The processed Seurat object was transformed into anndata format, following the SeuratDisk (v0.0.0.9011) pipeline, to facilitate RNA velocity analysis via Python. Subsequently, RNA velocity analysis was conducted via scVelo (v0.2.4). Following standard procedure using default parameters, data were filtered and normalized using scvelo.pp.filter_and_normalize, and first and second-order moments were computed with scvelo.pp.moments. Velocities were then calculated using scVelo’s dynamical model which solves the full transcriptional dynamics for each gene. A velocity graph was further computed to infer cell-to-cell transitions based on the vectors. The results were visualized via the original UMAP embedding method [17].

### Preprocessing and quality control of single-cell multiome data from a cell line-like model

We acquired multiomics data encompassing both scRNA-seq and scATAC-seq data. For preprocessing of the scRNA-seq data, we employed CellRanger software with default parameters to demultiplex the FASTQ reads for four distinct conditions: naïve, active, exhausted for 72 h and exhausted for 96 h. The reads were subsequently aligned to the mouse reference genome, and the cells were designated on the basis of UMI barcodes to generate single-cell gene count matrices. After cell filtration with a feature count threshold between 1,000 and 15,000, we utilized the SCTransform function to normalize the gene counts [10]. For scATAC-seq, we initiated the process by aligning reads to the mouse genome, thereby generating fragment files, through CellRanger ATAC. We then employed the Signac R package (v1.3.0) to compute the nucleosome signal strength and the transcription start site (TSS) enrichment score for each cell [18]. The cells were further filtered according to the following criteria: sequencing counts falling within the range of 1,000 to 150,000, nucleosome signal strength greater than 1.2, and TSS enrichment score exceeding 5. Then, we employed MACS2 (v2.2.7.1) to call peaks from the fragment files. For normalization of peak counts, we implemented term frequency-inverse document frequency (TF-IDF) normalization in Signac.

### Integration of single-cell multiome data from a cell line-like model

The Seurat objects from all the conditions were merged, and normalization was performed for both the RNA and chromatin assays. Partial singular value decomposition (SVD) was performed via the RunSVD function for latent semantic indexing (LSI) and dimensionality reduction [10]. To facilitate two-dimensional visualization, we utilized the weighted nearest neighbor method to construct a joint neighbor graph and project it on a two-dimensional plane using principal components 1 to 40 and LSI components 2 to 40 via UMAP.

### Multimodal reference mapping

To reveal the similarity between the in vivo and ex vivo model, we conducted multimodal reference mapping in Seurat using a three-step approach to map cells. First, we treated cells from the in vivo model as the reference and cells from the ex vivo model as the query, using the FindTransferAnchors function to compute the anchors between the reference and query. Next, we applied MapQuery to transfer cluster labels from the in vivo model to the ex vivo cells. Finally, we visualized the results on both UMAP and stacked bar plot to display the relationship between the in vivo and ex vivo model.

### Gene regulatory network inference

To infer the GRNs of T cells from both models, we employed the SCENIC R package (v1.2.0) [19]. SCENIC was utilized to identify highly activated GRNs associated with specific T-cell subtypes on the basis of scRNA-seq data. Initially, GRNBoost was employed to infer TFs and their target gene pairs on the basis of coexpression modules. Subsequently, candidate regulons were identified from coexpression modules by selecting direct-binding genes via the RcisTarget (v1.14.0) R package according to DNA motif analysis [19]. Area under the curve (AUC) scores were then calculated to quantify the relationships between TFs and downstream target genes with AUCell [19]. The GRN inference was performed independently on each of the two models. The resulting matrices of AUC scores were extracted and integrated with the Seurat object for subsequent analysis and visualization. To pinpoint significantly differentially expressed regulons, TFs were identified via the FindMarkers function in Seurat [10]. For downstream target gene selection, we compared the gene expression levels between exhausted CD8^+^ T cells and effector T cells according to the following criteria: log_2_FC > 0.25 and p value < 0.05.

### Functional enrichment analysis

AUCell was applied to identify the pathways that were enriched at the single-cell level. AUCell quantifies the enrichment level of a specified gene set by calculating the AUC based on a ranked gene list. For gene set collections from the mouse molecular signature database (MSigDB), the getGeneSets function in escape R package (v1.4.0) was utilized to obtain 50 hallmark gene sets and 185 curated gene sets derived from the Kyoto Encyclopedia of Genes and Genomes (KEGG) in the C2 collection. In practice, we ranked the genes on the basis of the normalized count matrices and calculated the AUC score for each gene set in each cell via the AUCell_calcAUC function with default parameters. The scores were then visualized in bar plots, depicting the fold enrichment between two groups. In addition, igraph (v1.2.6) was utilized for pathway visualization. Furthermore, to identify the biological functions of the Runx2 regulon, we conducted an over-representation analysis against the KEGG and GO Biological Process databases using the clusterProfiler R package (v4.2.2).

### Motif enrichment analysis

To reveal the relationship between the gene expression profile and DNA accessibility for the desired TF, we first conducted motif analysis to calculate the motif enrichment scores. Through Signac, we linked the normalized gene expression profiles with individual peaks by calculating the GC content and region lengths for each peak. This facilitated the exploration of the correlation between the identified peaks and the expression levels of nearby genes. For motif analysis, we initially acquired a matrix of vertebrate TF-binding motifs from the CORE collection of JASPAR2020 [20]. We subsequently identified the motif positions of each peak via the FindMotifs function in Signac, leveraging the R package motifmatchr (v1.16.0). Motif enrichment scores for each cell were then computed through the RunChromVAR function in Signac, utilizing chromVAR (v1.16.0). We also compared the normalized gene expression level and motif enrichment scores of each group to reveal the relationship between the DNA and RNA levels.

### Differentially accessible regions and gene activity

To identify the DARs in exhausted CD8^+^ T cells, we compared the peak counts between exhausted CD8^+^ T cells at 72 h and 96 h and active T cells through the FindMarkers function in Seurat with a logistic regression framework. To quantify the accessibility of each gene, we calculated the gene activity on the basis of the scATAC-seq data by computing counts per cell in the gene body and promoter region via Signac.

### Assessment of Runx2 expression in T cells in mice

To assess whether Runx2 was highly expressed and activated downstream targets in exhausted CD8^+^ T cells after anti-PD1 treatment, we used GEO dataset GSE174770, relevant to enhancing anti-PD1 efficiency in mice [21]. This dataset includes microarray data from 16 mice bearing subcutaneous HCC tumors and assigned to 4 different treatment groups. CD8^+^PD1^+^ T cells were collected from tumors on day 14 via flow cytometry and processed on a microarray platform. We extracted CD8^+^PD1^+^ T-cell microarray data from two conditions, placebo and anti-PD1 treatment, for external validation. The microarray data were preprocessed via the rma function from Affy (v1.72.0) and annotated via the Affymetrix HT_MG-430_PM database [22]. The microarray data were further analyzed via limma for differential expression with the lmFit and eBayes functions and visualized with a heatmap. Moreover, gene set variation analysis (GSVA) was conducted to estimate the variation in Runx2 and its downstream targets.

### Assessment of RUNX2 expression in T cells in HCC patients

To evaluate whether RUNX2 expression was elevated and correlated with the clinical outcome following anti-PDL1 treatment, we obtained transcriptomic data from 35 anti-PDL1 pretreated tumors, comprising 9 responders and 26 nonresponders, from the European Genome–Phenome Archive via the accession number EGAS00001005503 [23]. We utilized the DESeq2 R package (v1.34.0) for count matrix normalization. To extract CD8^+^ T-cell information from bulk RNA sequencing data, we scaled the matrix on the basis of the *CD8A* expression level in each sample. To determine the relationship between the RUNX2 module and the clinical outcome, we conducted GSVA to estimate the variation in Runx2 and its downstream targets and compared the modules active in responders and nonresponders.

### Assessment of RUNX2 expression in T cells in HCC patients after anti-PD1 treatment

To evaluate whether RUNX2 was highly expressed and correlated with the clinical outcome following anti-PD1 treatment, we used spatial transcriptomics data from 8 anti-PD1-treated HCC patients, including five nonresponders and three responders collected from Mendeley Data (skrx2fz79n) [24]. We utilized Seurat for preprocessing, clustering the eight spatial transcriptomics samples independently and reannotating the cell types according to the expression profiles of selected well-known marker genes (Table S1). To increase the resolution for the desired genes, we conducted a series of analyses by using BayesSpace (v1.4.1). The spots were first enhanced through the spatialEnhance function, and the expression profiles of the desired genes were enhanced through the enhanceFeatures function (Table S2). The enhanced features of each subspot were averaged and assigned back to the original spots. We then normalized the enhanced feature profile and calculated the module scores, including those of the RUNX2 module and exhausted T-cell module, by averaging the normalized values (Table S2). Additionally, we manually delineated the carcinoma region and quantified the number of CD8^+^ T-cell spots as spots with CD8A and CD3D or CD3E expression levels above their respective 75th percentiles. To reveal the relationship between the RUNX2^+^ exhausted T-cell population and clinical anti-PD1 treatment outcomes, we calculated and compared the proportions of RUNX2^+^ exhausted CD8^+^ T cells relative to CD8^+^ T cells in responders and nonresponders.

### Assessing the effect of CADD522 on T-cell function

To explore the impact of Runx2 on the progression of CD8^+^ T cells, we treated these cells ex vivo with the Runx2 inhibitor CADD522 [25]. CADD522 was purchased from Selleckchem Corporation (Houston, TX). We subjected CD8^+^ T cells derived from P14 splenocytes to repeated stimulation with the gp33 peptide to induce exhaustion in the presence of CADD522 (100 µM) or vehicle control for 96 h. We performed a cytotoxicity assay and analyzed marker expression via BD LSRFortessa Cell Analyzer and FlowJo software (v10.10). The flow cytometry and cytotoxicity assay protocols were performed as described above. In addition, we observed the viability of Hepa1‒6 liver cancer cells treated with different concentrations of CADD522. We plated 1000 cells in 96-well plates and treated them with CADD522 (100 µM) or the vehicle control for 96 h. Then, we processed the MTT assay by adding 50 µl of 2 mg/ml thiazolyl blue (MedChemExpress) for 4 h in a 37 °C incubator. We discarded the supernatant and measured the absorbance at OD = 540 nm after adding 150 µl of DMSO (Sigma‒Aldrich). We defined the value observed in Hepa1-6 cells treated with 0 µM CADD522 as 100% cell viability as the reference for calculation of cell viability after treatment with different concentrations of CADD522.

### Statistical analysis

All the statistical analyses were performed via the stats (v4.1.3) R package, and graphs were generated via the ggplot2 (v3.3.5) and ComplexHeatmap (v2.10.0) R packages. The data are presented as the means ± SEs from biological replicates. For comparisons between two groups, unless otherwise specified, the F test was used to compare the variances of the two samples, followed by the Mann-Whitney U test to assess statistical significance. A p value of less than 0.05 was considered to indicate statistical significance. Detailed statistical analyses and the number of biological replicates from independent experiments are reported in the figure legends.

## Results

### ScRNA-seq profiling of T cells from the HCC mouse model

**Fig. 1:**
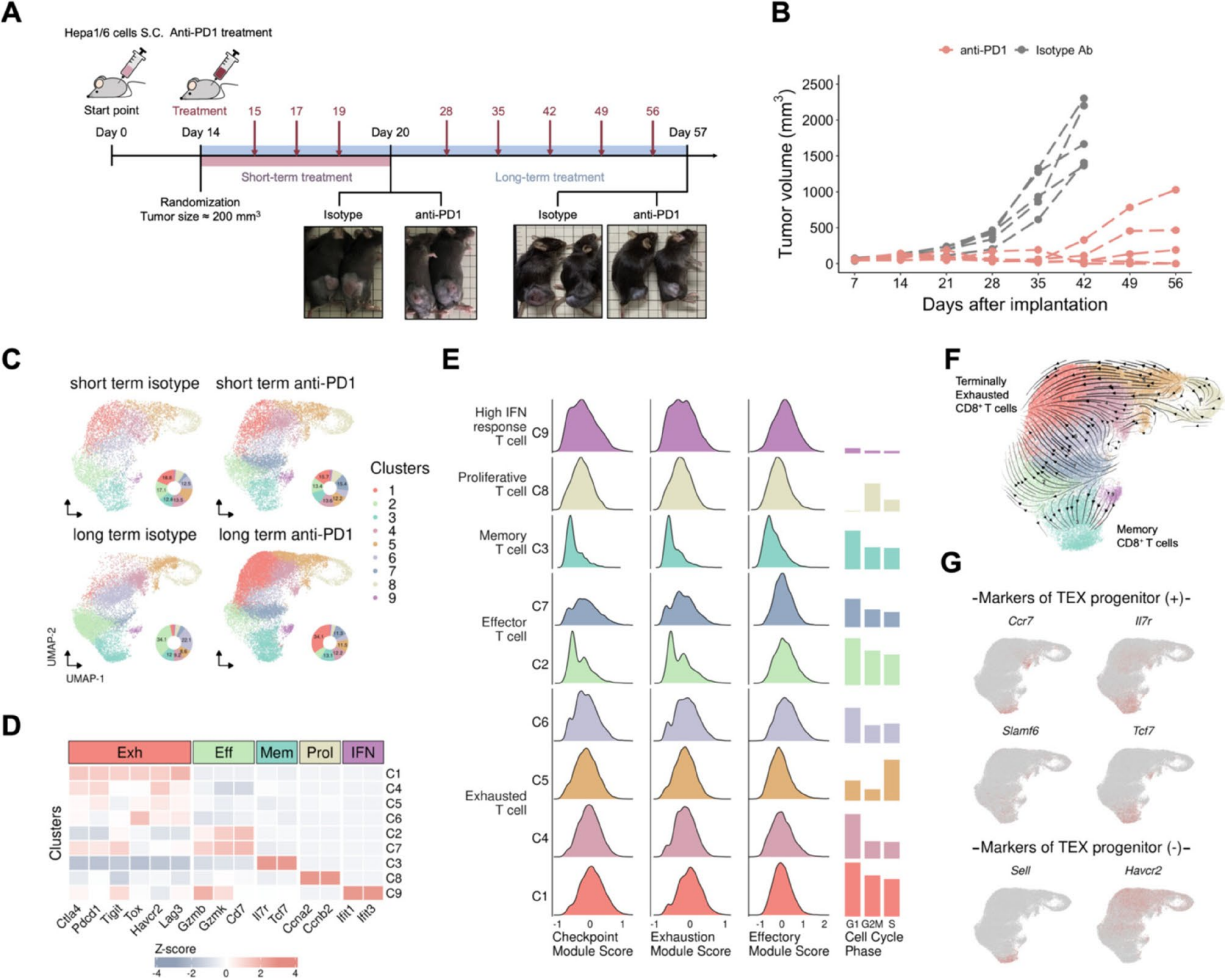
Single-cell RNA-seq profiling of CD8^+^ T cells from an in vivo model. **A** In vivo mouse model schema: We subcutaneously implanted Hepa1-6 mouse hepatocellular carcinoma cells into C57BL/6 mice. After 14 days, the mice were randomized and subjected to short-term (20 days) and long-term (57 days) treatments to collect T cells and tumor samples after anti-PD1 or isotype treatment. The samples were extracted from tumor cells and CD8 + T cells for single-cell RNA sequencing. **B** Tumor growth results from the in vivo mouse model. The data show the response to anti-PD1 and isotype control treatment. Three mice developed acquired resistance to long-term anti-PD1 treatment from a group of five. **C** UMAP embedding of the cell composition of the four groups from the mouse model. Cluster 1 (exhausted CD8^+^ T cells) was enriched in the long-term anti-PD1 treatment group, and cluster 2 (effector T cells) was enriched in the long-term isotype group. **D** Heatmap showing the expression profiles of select markers in each T-cell cluster. According to these data, we categorized the nine clusters into five distinct T-cell subtypes: exhausted, effector, memory, proliferative and high interferon (IFN) response. **E** Runx2 inhibitors improved the effector function of exhausted CD8^+^ T cells. Ridge plots reveal three self-defined module score differences between each cluster, where exhausted CD8^+^ T cells have higher checkpoint and exhaustion module scores but lower effector module scores. Bar plots depict the composition of each cell cycle phase within each cluster. **F** RNA velocity analysis reveals bifurcated T-cell differentiation trajectories toward terminally exhausted and memory CD8^+^ T-cell fates. **G** Cells are colored according to the expression level of exhausted T-cell progenitor (+) or (-) marker genes, which indicated that exhausted T-cell progenitors belong to cluster 4

To elucidate the mechanisms of progressive T-cell exhaustion underlying acquired resistance to ICIs, we initially developed an in vivo model by subcutaneously implanting Hepa1-6 HCC cells into mice and subsequently treating them with either an anti-PD1 antibody or an isotype control (Fig. 1A). The tumor size increased sharply in the control group. In contrast, the anti-PD1 group showed an initial reduction in tumor size, implying a good response to anti-PD1 treatment. However, over prolonged treatment periods, three mice in the anti-PD1 group developed acquired resistance to anti-PD1 treatment, leading to incomplete tumor elimination and regrowth (Fig. 1B). The emergence of a resistant phenotype in our in vivo model set the stage for further exploration into the molecular mechanisms governing T-cell exhaustion, which in turn causes ICI resistance. We collected tumor samples from four groups, namely, the short-term anti-PD1, isotype control, long-term anti-PD1 and isotype control groups, to study the mechanism of CD8^+^ T-cell exhaustion. The short-term anti-PD1 groups presented a good response to immunotherapy, and the long-term anti-PD1 groups presented acquired resistance to anti-PD1 treatment. To gain a comprehensive understanding of the progression of T-cell exhaustion under acquired resistance, we used cell sorting to collect CD45^−^ and CD45^+^CD8^+^ cells and conducted scRNA-seq to elucidate the process of T-cell exhaustion (Table S3). This dual-timepoint approach allowed us to discern dynamic changes in CD8^+^ T cells, providing insights into both the initial response to ICI therapy and the subsequent development of resistance over an extended treatment duration. We performed data preprocessing, unsupervised clustering and two-dimensional projection, and the cells were distinguished into 11 distinct clusters (Fig. S1A). To extract the CD8^+^ T-cell populations from the mixed of CD45^−^ and CD45^+^CD8^+^ cells in our scRNA-seq data, we separated the CD8^+^ T cells and tissue cells via two well-known CD8^+^ T-cell markers, *Cd3e* and *Cd8a*, from the mice (Fig. S1B-C). On the basis of the expression levels of T-cell marker genes, six clusters of cells were defined as CD8^+^ T cells, and the remaining clusters were assigned to tumor tissues.

### Identification of exhausted CD8^+^ T cells from an in vivo model

Following the isolation of CD8^+^ T-cell populations, we applied a reprojection and reclustering approach, resulting in the identification of nine distinct clusters (Fig. 1C). The cell cluster and subtype composition varied significantly among samples from distinct experimental conditions (Fig. S2). To characterize and annotate the CD8^+^ T-cell subtypes, we curated a set of transcriptomic markers associated with specific T-cell subpopulations from previous studies [14, 26, 27]. Each cluster was examined for the expression of the following T-cell-related marker genes: exhausted CD8^+^ T cell markers (Ctla4, Havcr2, Lag3, Pdcd1, Tigit), effector T cell markers (Gzmb, Gzmk, Cd7), memory T cell markers (Il7r, Tcf7), proliferating T cell markers (Ccna2, Ccnb2) and interferon (IFN)-responsive T cell markers (Ifit1, Ifit3) (Fig. 1D, Fig. S3A-B). Based on marker expression levels, we annotated five different CD8^+^ T-cell subtypes (Fig. S3C). Through the analysis of the subtype proportions within each experimental condition, a notable trend in T-cell composition was observed, particularly in the long-term treatment group (Fig. S3D). Compared with those in the other three conditions, the exhausted CD8^+^ T cells clearly increased in terms of both absolute numbers and relative proportions.

In addition to our analysis of T-cell subtypes based on biomarker expression, we further enriched our understanding of T-cell functionality by designing a comprehensive set of functional gene modules (Table S4). We calculated the module score and presented the result in a ridge plot with modules comprising groupings of genes with similar functions, providing a systematic and functional perspective on the diverse roles of T cells (Fig. 1E). Given that cluster 1 consists of exhausted CD8^+^ T cells, its notable increase within the long-term anti-PD1 group—coupled with its high expression of checkpoint and exhaustion modules—raises intriguing possibilities about the potential impact on the efficacy of ICI treatment and resistance.

### Differentiation dynamics analysis reveals the T-cell exhaustion process

Numerous studies have shown that T cells transition from the resting state to the activated state in response to ICI treatment and regain their cytotoxic function to eliminate cancer cells [28–31]. However, with prolonged ICI treatment, T cells might undergo a progressive exhaustion process, thus developing acquired resistance. To reveal the process of exhausted CD8^+^ T-cell differentiation, we performed RNA velocity analysis and detected distinctive convergence patterns among T cells, which were primarily consolidated into two predominant cell populations (Fig. 1F, Fig. S4). The first group included memory T cells, which represent an end-stage subtype involved in the T-cell differentiation process. Conversely, dynamic modeling indicated convergence toward a unique subset of exhausted CD8^+^ T cells, predominantly cluster 1 cells. This exhausted T-cell population manifested over an extended period of ICI treatment and therefore was a significant focal point of our investigation. In our pursuit of elucidating the path of progressive CD8^+^ T-cell exhaustion, we also focused our attention on several specific markers associated with T-cell exhaustion progenitors (Fig. 1G). We found that these exhausted T-cell progenitors are identified within cluster 4. To further delineate these progenitor subsets, subclustering analysis was conducted, which revealed a prominent progenitor subcluster, specifically denoted c4_4, residing within cluster 4 (Fig. S4). This specific subpopulation, c4_4, would be particularly worth further study as it represents a prominent progenitor subcluster related to immunotherapy resistance. The identification of T-cell exhaustion progenitors contributed to mapping the trajectory of T-cell exhaustion, offering valuable insights into the process that leads to the development of exhausted CD8^+^ T cells and results in ineffective suppression of tumor growth and the prognosis of ICI therapy. Further exploration of the mechanism underlying the T-cell exhaustion process during ICI treatment within cluster 4 and cluster 1 could pave the way for targeted interventions aimed at enhancing the effectiveness of immunotherapeutic strategies.

### Single-cell multiome analysis of externally stimulated T cells from an ex vivo model

**Fig. 2:**
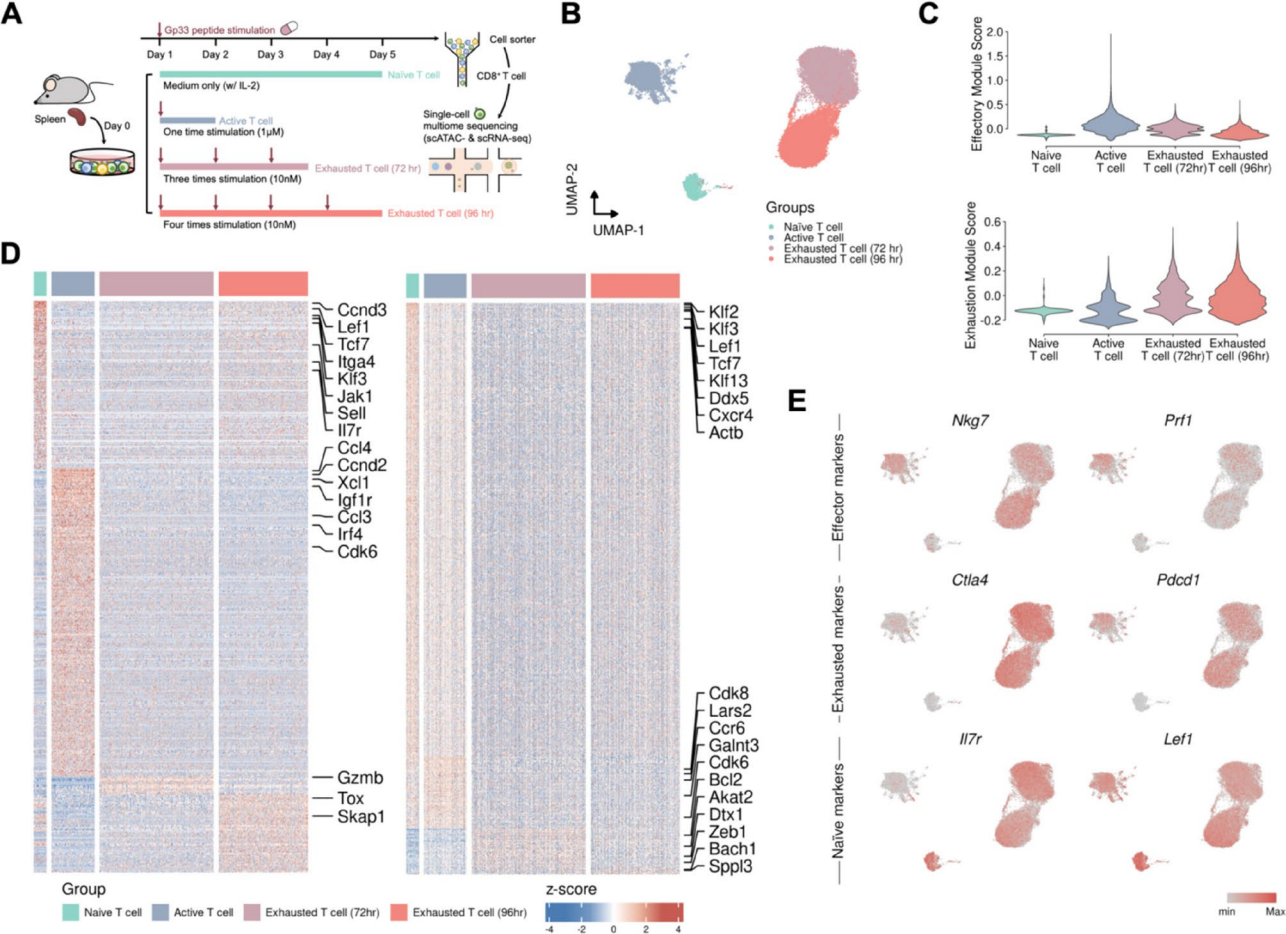
Single-cell multiome sequencing profiling of CD8^+^ T cells from an ex vivo model. **A** Ex vivo mouse model schema: We isolated splenocytes from P14 mice, whose CD8^+^ T cells recognize the gp33 peptide, to generate CD8^+^ T cells of various induced statuses. Naïve CD8^+^ T cells were cultured for four days with only 100 U of IL-2 added. Active and exhausted CD8^+^ T cells were stimulated with the gp33 peptide: high-dose (1 µM) for 24 h for active cells and low-dose (10 nM) repeatedly for 72–96 h for exhausted cells. We subsequently sorted live CD8^+^ T cells for single-cell RNA sequencing. **B** UMAP embedding of integrated multiome data. **C** Violin plot of the module scores of the effector and exhaustion modules. **D** Heatmap of the expression levels of the top 10 highly variable genes in each group. **E** Cells are colored according to the expression levels of well-known T-cell functional marker genes, including effector markers (Nkg7 and Prf1), exhaustion markers (Ctla4 and Pdcd1) and naïve markers (Il7r and lef1)

To create a spectrum of T-cell states, we designed an ex vivo model in which cultured P14 T cells were stimulated with the gp33 peptide, which is specifically recognized by CD8^+^ T cells from these P14 mice, with different stimulation concentrations and frequencies and culture durations (Fig. 2A). To identify the induction statuses of CD8^+^ T cells ex vivo, we detected surface markers and transcription factors by flow cytometry and conducted a cytotoxicity assay with different E-T ratios (Fig. S6). We conducted single-cell multiome sequencing, including scRNA-seq for the transcriptome and scATAC-seq for chromatin accessibility, to identify crucial exhaustion regulatory factors and gain deeper insights into T-cell exhaustion mechanisms. For single-cell multiome data analysis, we first integrated the transcriptome data with chromatin accessibility data and projected them onto a two-dimensional plane (Fig. 2B). Two exhausted T cell clusters projected closely together while remaining distantly separated from both naïve and active T cells. First, we determined the expression levels of specific T-cell subtype markers and scores of functional modules where active T cells presented elevated scores in the effector module, indicative of increased effector functions, whereas the two exhausted CD8^+^ T cell samples presented increased scores in the exhaustion module, illustrating their exhaustion state (Fig. 2C). In addition, to examine the characteristics of T cells, we conducted differential expression analysis and differential accessibility analysis to identify significantly differentially expressed genes and differentially accessible regions in each group and generated a heatmap (Fig. 2D). Our findings revealed that both the differentially expressed genes and the differentially accessible regions were more similar between the two exhausted T-cell groups than between these and other T-cell groups, indicating a degree of similarity in their transcriptional responses. In contrast, naïve and active T cells presented more distinct expression and accessibility patterns. This finding suggested that T cells subjected to multiple types of stimulation gradually progress toward exhaustion, resulting in a convergence of their transcriptional profiles over time. The distinct gene expression patterns observed in naïve and active T cells further support the notion that repeated stimulation induces a unique transcriptional signature associated with T-cell exhaustion that coincides with the loss of cytotoxic functionality. We also revealed the expression level of well-known T-cell related marker genes to verify the T-cell state (Fig. 2E). Additionally, we conducted reference mapping between two distinct models. Using the in vivo model as a reference, we mapped T cells from the ex vivo model to each in vivo cluster (Fig. S7A). Additionally, we calculated the proportion of each condition in the ex vivo model, finding that two exhausted groups were primarily composed of exhausted CD8^+^ T cells, while naïve T cell group was consisted of memory T cells from the in vivo model. Moreover, we observed that the exhausted CD8^+^ T cells within the active T cell group belonged to cluster 4 in the in vivo model, suggesting that cluster 4 marked the onset of CD8^+^ T-cell exhaustion trajectory (Fig. S7B-C). Consequently, we focused on both cluster 1 and 4 from the in vivo model for further analysis, as they represented the two endpoints of the CD8^+^ T-cell exhaustion process.

### Internal mechanisms regulating CD8^+^ T-cell exhaustion in acquired resistance

**Fig. 3:**
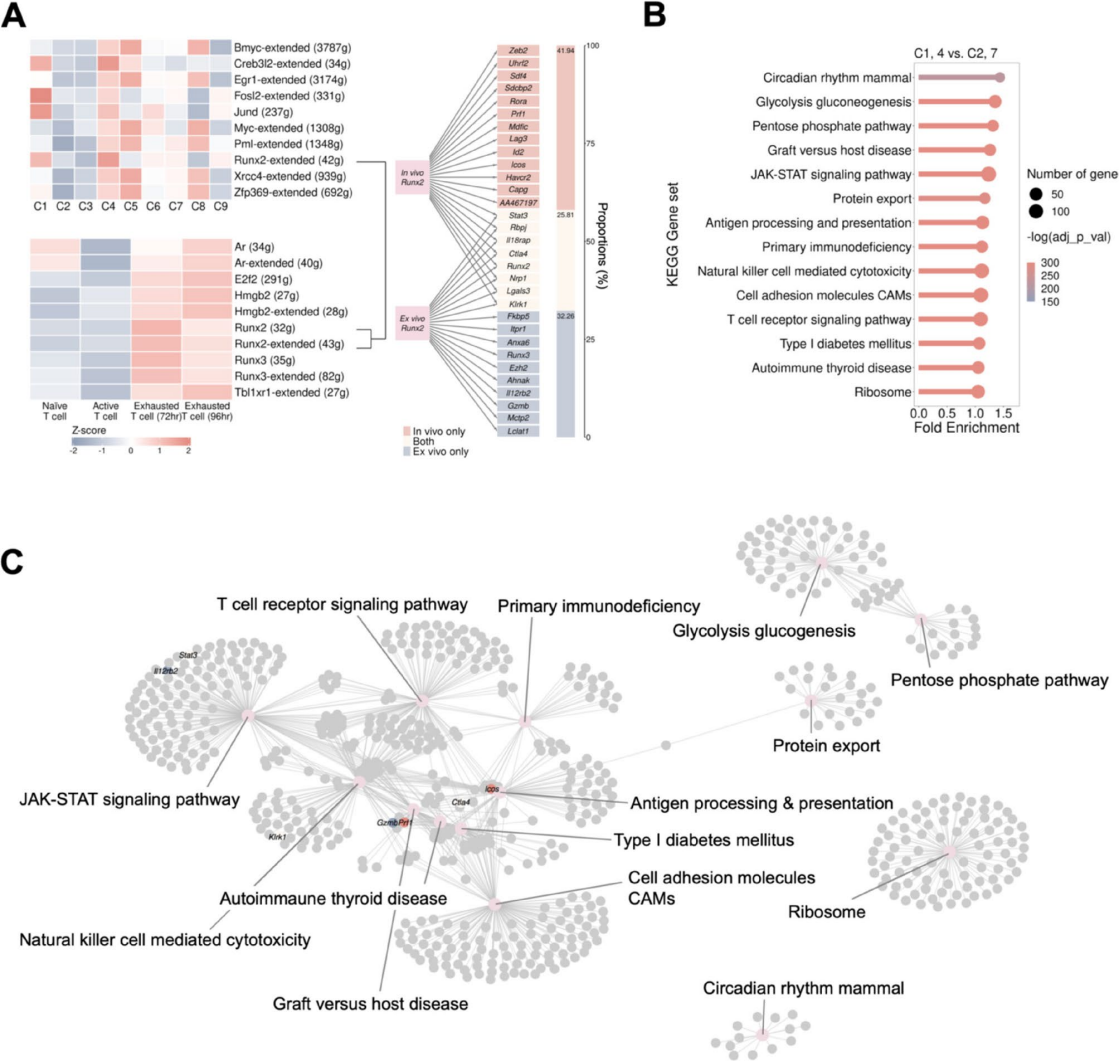
Transcription factor prediction and pathway enrichment analyses.** A** Exhausted CD8^+^ T cells from both in vivo and ex vivo models expressed high levels of Runx2 during exhaustion progression, as determined via transcription factor prediction. Downstream target genes of Runx2 with significantly high expression levels in exhausted CD8^+^ T cells from both models were revealed and used to construct the bipartite network. **B** We conducted gene set enrichment analysis using KEGG database via AUCell, and pathways were selected on the basis of differential expression (fold enrichment > 1) and significant expression level (adjusted p value < 0.05). **C** Network visualization revealed the relationships between the significantly highly expressed pathways and the Runx2 regulon, where several genes were identified and collected across various pathways

To identify the GRN perturbations and cellular signaling disruptions leading to CD8^+^ T-cell exhaustion in acquired resistance to ICIs or upon external stimulation, we predicted potential transcription factors and their downstream regulatory networks. We focused on identifying unique cellular network regulators, specifically, TFs that were highly expressed in CD8^+^ T cells during the induction of exhaustion by ICI treatment or external stimulation. Our attention was directed toward TFs that were associated with the trajectory from cluster 4 to cluster 1 from the in vivo model and two exhausted T-cell groups from the ex vivo model. The identification of these notable TFs provides valuable insights into the transcriptional regulation of CD8^+^ T-cell exhaustion, offering potential targets for further investigation and therapeutic modulation. For TF prediction, we utilized SCENIC on both in vivo and ex vivo models independently (Fig. S8-9). The comparative analysis of the top 10 TFs between the two models revealed that only one TF, Runx2, demonstrated high expression in the development of CD8^+^ T-cell exhaustion, including in clusters 1 and 4 in the acquired resistance model, as well as in the two exhausted T-cell groups from the ex vivo model (Fig. 3A). The marked presence of this TF in the transcriptional profiles of multiple clusters of exhausted CD8^+^ T cells suggested its potential importance in orchestrating the regulatory networks associated with the CD8^+^ T-cell fate decision. Furthermore, to construct the complete Runx2 regulatory network of the progressive T-cell exhaustion process, we identified 31 downstream targets, including eight genes that were highly expressed in exhausted CD8^+^ T cells from both models, revealing the potential molecular mechanisms governing CD8^+^ T-cell exhaustion after ICI treatment. Additionally, to elucidate the biological pathways activated in exhausted CD8^+^ T cells after ICI treatment, we performed pathway enrichment analysis via the KEGG database. The results highlighted fourteen significantly upregulated pathways, including those related to primary Immunodeficiency, in clusters 1 and 4 in comparison with those in effector T cells (Fig. 3B). Building on our exploration of the Runx2 regulon and the enriched pathways, we further dissected the interconnections between Runx2 and these pathways. By overlapping the gene sets of the significantly upregulated pathways in exhausted CD8^+^ T cells with the targets regulated by Runx2, we identified several genes that were shared between these sets (Fig. 3C). To define the biological functions of the Runx2 regulon, we also performed an over-representation analysis (ORA) using Gene Ontology (GO) and KEGG databases. This analysis revealed that Runx2 target genes are significantly enriched in pathways related to T-cell activation, cell killing and cell-cell adhesion (Fig. S10). These observations suggested that the Runx2 regulon indeed exerted a regulatory influence on the functionality of CD8^+^ T cells, specifically within the context of progressive CD8^+^ T-cell exhaustion.

### Transcriptomic regulation of Runx2 in T-cell exhaustion

**Fig. 4:**
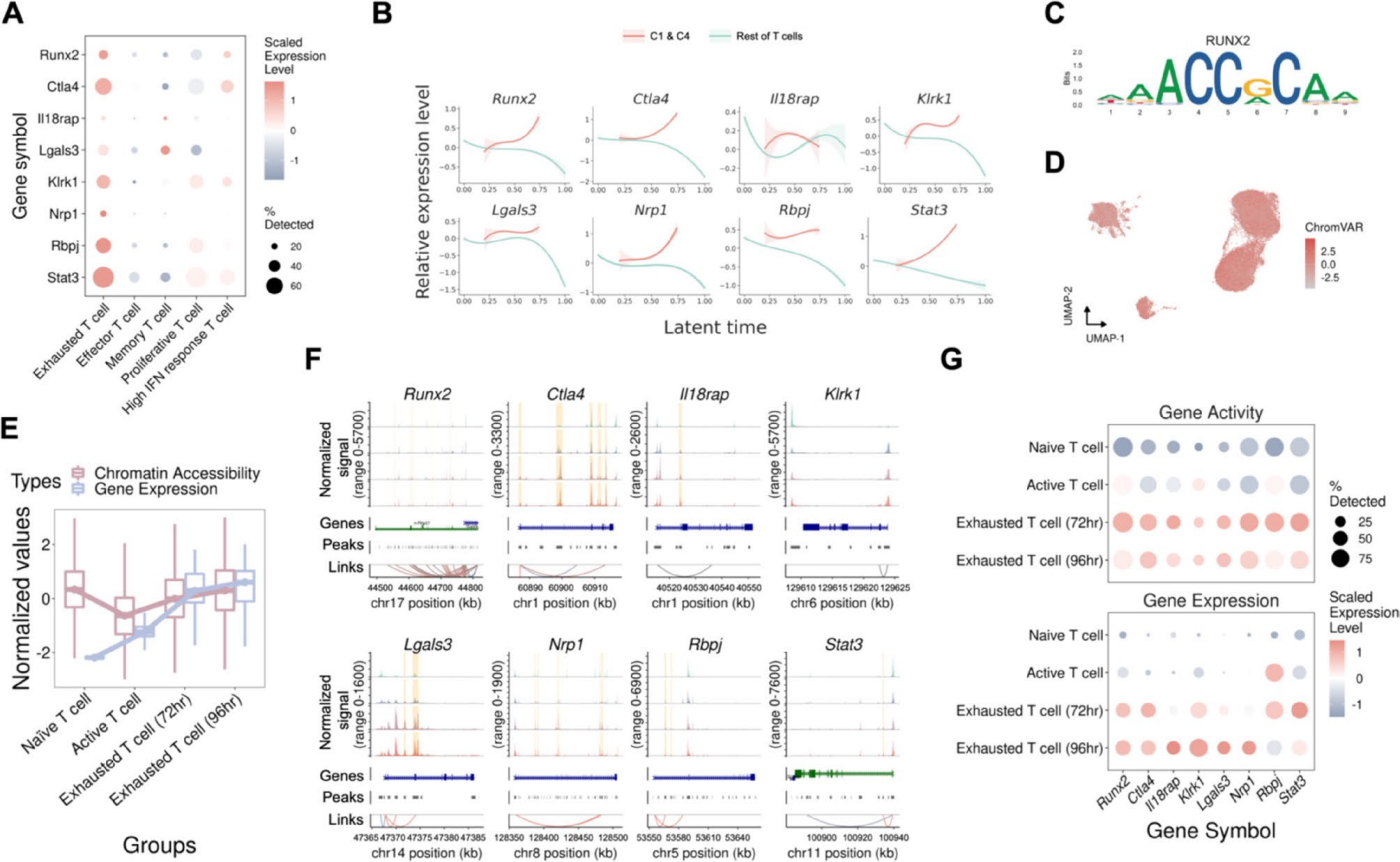
Characteristics of Runx2 and its downstream targets in both models. **A** Dot plot illustrating the gene expression patterns of Runx2 and its downstream targets. **B** Scatter plots showing the dynamic expression of Runx2 and its downstream targets over pseudotime. **C** Runx2 motif binding site. **D** The motif activity level was calculated via chromVAR with UMAP embedding. **E** Runx2 motif activity in each group. **F** Chromatin accessibility of Runx2 and its downstream targets in each group of cells. Regions with significantly increased chromatin accessibility in exhausted CD8^+^ T cells (72 h and 96 h) compared with those in active T cells are highlighted in orange. **G** Dot plot illustrating the gene activity and expression patterns of Runx2 and its downstream targets, demonstrating a consistent pattern between gene activity and gene expression

As Runx2 was found to be a significantly active TF in the entire CD8^+^ T-cell exhaustion process after ICI treatment, we first revealed the high expression level of Runx2 regulon (comprising Runx2 and its identified downstream target genes) in exhausted T cells, especially cluster 1 and 4, from the in vivo model (Fig. 4A, Fig. S11A). In our pursuit of deeper insights into the role of Runx2 in T-cell exhaustion, we calculated the Runx2 signature score and revealed the relationship between the Runx2 signature score and the T-cell exhaustion signature score (Fig. S11B-E). This positive correlation and the results of previous analyses led us to believe that Runx2 is an important TF involved in progressive T-cell exhaustion related to acquired resistance. Building on these findings, we performed in-depth analysis of the expression dynamics of the Runx2 regulon over time. This investigation revealed that anti-PD1 resistance-related exhausted CD8 + T cells demonstrated progressively higher expression levels of Runx2 and its regulon members throughout the exhaustion process (Fig. 4B). This exploration revealed that the expression levels of Runx2 target genes were elevated in clusters 1 and 4 and tended to increase with increasing latency time. Using ChromVAR, we calculated the Runx2 motif activity for each group and subsequently explored the relationship between motif accessibility and Runx2 gene expression (Fig. 4C-E). The results indicated that the Runx2 motif was more accessible and expressed in exhausted CD8^+^ T cells than in active T cells. This observation underscores the association between Runx2 motif accessibility and gene expression in the context of progressive T-cell exhaustion. The elevated motif accessibility in exhausted T-cell groups suggests the active involvement of Runx2 in the regulatory landscape of these cells, further reinforcing its potential role in driving the transcriptional changes associated with T-cell exhaustion. Further, we investigated the chromatin accessibility of each Runx2 target gene (Fig. 4F). This analysis revealed a substantial number of regions with heightened accessibility specifically in exhausted CD8^+^ T cells, which suggested that Runx2 contributes to shaping the epigenetic landscape during the progression of T-cell exhaustion. Finally, we conducted an analysis of both gene activity and gene expression levels for each member of the Runx2 regulon (Fig. 4G). The result revealed a consistent pattern, indicating that all the members of the Runx2 regulon were highly activated in exhausted CD8^+^ T cells. This alignment between gene activity and expression further emphasized the concerted and heightened regulatory impact of Runx2 on its downstream targets during T-cell exhaustion. In summary, our comprehensive analyses revealed the central role of Runx2 in progressive T-cell exhaustion related to acquired resistance. It was not only highly expressed in exhausted CD8^+^ T cells but also demonstrated downstream regulatory activity with increased accessibility to specific motifs, influencing the T-cell exhaustion process. Recognizing the potential impact of Runx2 on T-cell exhaustion, we identified specific targets within the Runx2 regulon. We subsequently pursued the discovery of direct inhibitors for these selected targets to improve the efficiency of ICIs by preventing T cells from entering the exhaustion process (Table S5).

### Expression of Runx2 in T cells associated with the efficacy of ICI therapies

**Fig. 5:**
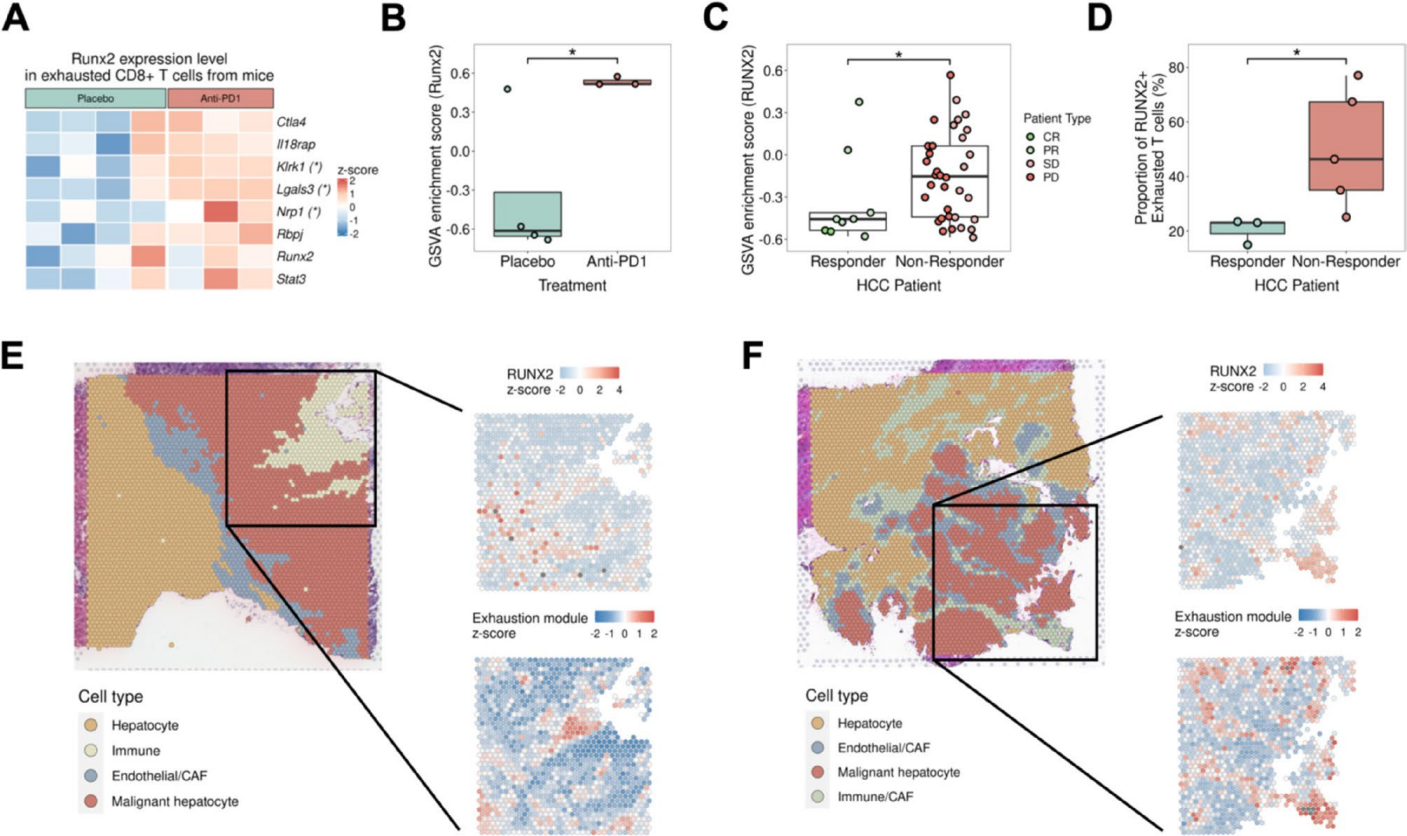
Investigation of Runx2 regulon enrichment in three external datasets. **A** Heatmap showing that exhausted CD8^+^ T cells in the anti-PD1 treatment group expressed high levels of Runx2 and its downstream targets using the mice microarray data from GEO dataset (GSE174770). **B** According to GSVA on the mice microarray data from GEO dataset (GSE174770), exhausted CD8^+^ T cells from the anti-PD1 treatment group (n = 4) had significantly higher Runx2 GSVA enrichment scores than did those from the placebo group (n = 3). **C** Box plot showing the significant difference in the RUNX2 GSVA enrichment score between nonresponders (n = 26) and responders (n = 9) via Wilcoxon rank sum test using the clinical data from the European Genome–Phenome Archive via the accession number EGAS00001005503. **D** Analysis of the spatial transcriptomics dataset from Mendeley data (skrx2fz79n) revealed that the proportion of RUNX2^+^ exhausted CD8^+^ T cells was significantly greater in nonresponders (n = 5) than in responders (n = 3) to anti-PD1 therapy. **E** A representative ICI responder spatial transcriptomics dataset was reannotated (left panel), and the Runx2 expression level and exhaustion module score of the carcinoma region were determined (right panel), which revealed a strong correlation between the Runx2 expression level and the exhaustion module score in immune cells. **F** A representative ICI nonresponder spatial transcriptomics dataset was reannotated (left panel), and the Runx2 expression level and exhaustion module score of the carcinoma region were determined (right panel), which revealed a weak correlation between the Runx2 expression level and the exhaustion module score in immune cells. Complete response (CR), partial response (PR), stable disease (SD) and progressive disease (PD), *p < 0.05

Our data revealed the role of Runx2 in the progression of T-cell exhaustion after anti-PD1 treatment or external stimulation. Next, we sought to validate our findings in three external datasets (Fig. S12A). We first analyzed the in vivo microarray data from GEO (GSE174770). We extracted CD8^+^ PD-1^+^ T-cells from mice with subcutaneous tumors treated with either a placebo or anti-PD1 therapy. We found that all the members of the Runx2 regulon were expressed at higher levels in the anti-PD1 antibody-treated group than in the placebo group (Fig. 5A). Furthermore, we employed GSVA to assess the enrichment scores of the Runx2 regulon in exhausted CD8^+^ T cells subjected to anti-PD1 treatment and compared them to those of the placebo group (Fig. 5B). The results of GSVA revealed a significant increase in the enrichment score of the Runx2 regulon in exhausted CD8^+^ T cells treated with anti-PD1, indicating robust and systematic upregulation of the Runx2-related pathway in response to the exhaustion process with ICIs. Next, we analyzed RNA-seq data from pre-treatment HCC patients (EGAS00001005503). We specifically extracted information related to CD8^+^ T cells, performed GSVA, calculated the enrichment score of the Runx2 regulon and revealed that the enrichment score was significantly greater in nonresponders than in responders (Fig. 5C, Fig. S12B). Moreover, we analyzed the spatial transcriptomics data of HCC patients receiving anti-PD1 therapy, collected from Melendey data (skrx2fz79n). Through cell type annotation and manual demarcation of the carcinoma region, we quantified the proportion of RUNX2^+^ exhausted CD8^+^ T cells. Strikingly, our findings revealed a greater proportion of the RUNX2^+^ exhausted T-cell subset in nonresponders than in responders (Fig. 5D). The observed increased proportion in nonresponders verified the association between the abundance of RUNX2^+^ exhausted CD8^+^ T cells and response to anti-PD1 therapy in HCC patients. Moreover, we revealed that the expression patterns of the RUNX2 and exhaustion modules were similar in nonresponders but varied in responders (Fig. 5E-F, Fig. S13). In summary, the results of external validation were consistent with the previously observed phenomenon, providing further information about the association between elevated regulation of RUNX2 and a lack of response to ICI treatment. These results underscore the potential clinical importance of targeting RUNX2 transcriptional regulation to enhance treatment responses in HCC patients undergoing immunotherapy.

### Runx2 inhibitors improved the effector function of exhausted CD8^+^ T cells

**Fig. 6:**
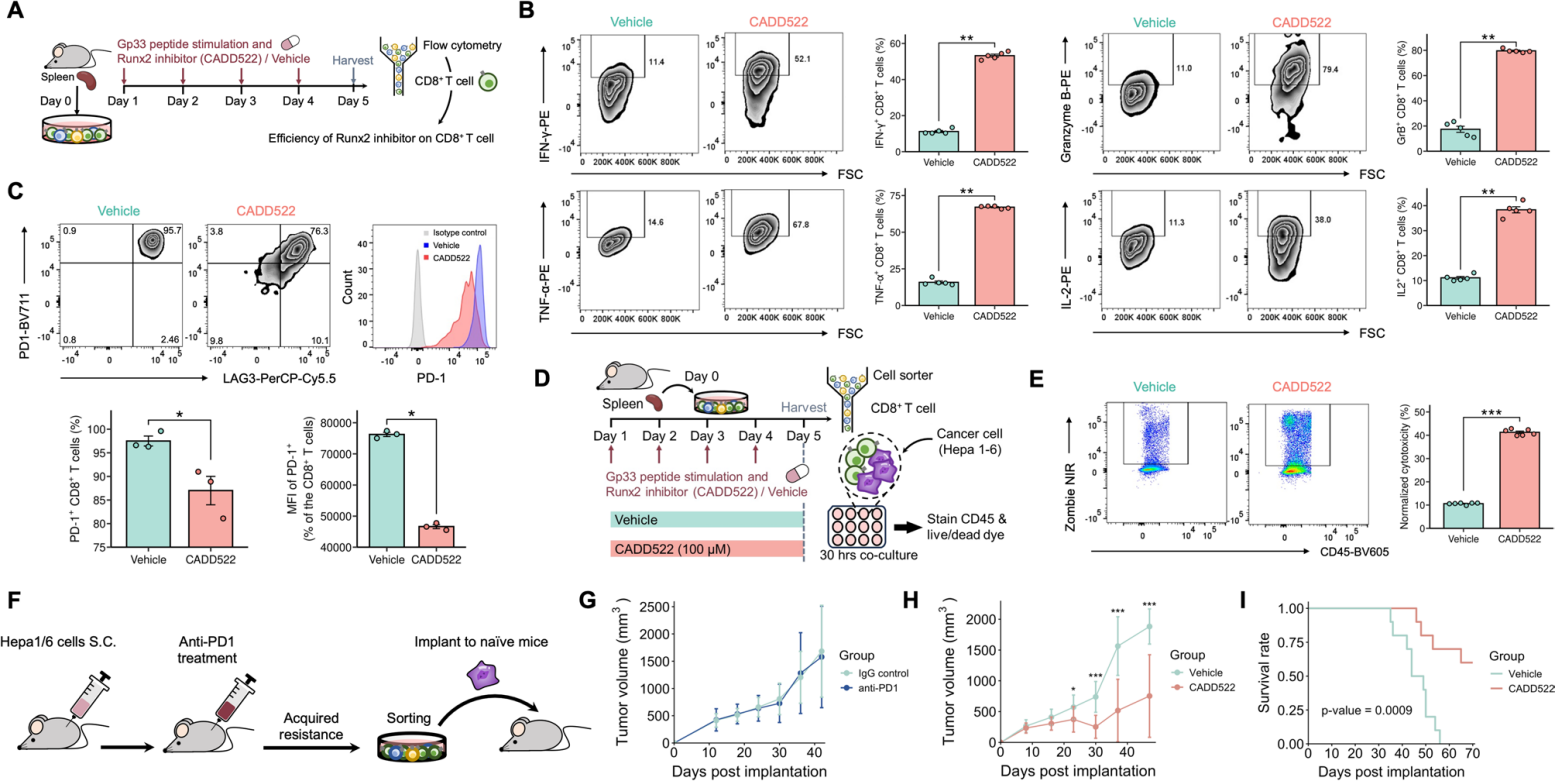
Runx2 inhibitors improved the function of exhausted CD8+ T cells. **A** Experimental schema: Ex vivo model exhausted CD8+ T cells were treated with the Runx2 inhibitor CADD522 (100 μM) or vehicle control. After treatment, cells were harvested for flow cytometry analysis. **B** Detection of functional markers in exhausted CD8+ T cells treated with Runx2 inhibitor or vehicle (n = 5). **C** PD-1 expression in an ex vivo model of exhausted CD8+ T cells treated with Runx2 inhibitor or vehicle. The histogram below showed less PD-1+ CD8+ T cells after treatment with Runx2 inhibitor (n = 3). **D** Experimental schema:Exhaustion was induced in P14 CD8+ T cells by repeated stimulation with gp33 in the presence of either Runx2 inhibitor or vehicle for 96 h. These cells were then cocultured with Hepa1-6 gp33 cancer cells at a 1:1 effector-to-target ratio for 30 h, followed by detection of the CD45 marker and live/dead dye via flow cytometry after the cells were stained. **E** Exhausted CD8+ T cells treated with Runx2 inhibitor or vehicle, then coculturing with Hepa1‒6 gp33 cancer cells (n = 6). The viability assay used the spontaneous death of Hepa1-6 gp33 cancer cells as the baseline control. **F** Schematic of establishing an anti-PD1-resistant Hepa1-6 cell line through in vivo selection and reimplantation. **G** Tumor growth curves of the resistant Hepa1-6 cell line implanted into naïve C57BL/6 mice treated with either anti-PD1 antibodies or IgG control (200 μg/intraperitoneal, ×5). Tumor growth showed no significant response to anti-PD1 treatment, confirming the resistant phenotype (n = 5/group). **H** Tumor growth curves of resistant Hepa1-6 tumors treated with the RUNX2 inhibitor CADD522 (100 μM) or vehicle control. CADD522 treatment resulted in reduced tumor growth compared to vehicle (n = 10/group). **I** Kaplan–Meier survival curves of mice bearing resistant Hepa1-6 tumors treated with CADD522 or vehicle control (n = 10/group). *p < 0.05; **p < 0.01; ***p < 0.001; ****p < 0.0001, as assessed via two-tailed Student’s t test. Data are presented as mean ± standard error (SE)

To explore the impact of Runx2 on the progression of CD8^+^ T-cell exhaustion, we treated these cells ex vivo with the small-molecule Runx2 inhibitor CADD522. Previous research has shown that CADD522 inhibits growth in breast cancer cells and improves outcomes in a xenograft mouse model of bone cancer by targeting Runx2 [32]. In our study, we treated exhausted CD8^+^ T cells derived from P14 splenocytes with repeated stimulation with the gp33 peptide in the presence of the Runx2 inhibitor CADD522 or the vehicle control for 96 h (Fig. 6A). We then assessed marker expression on CD8^+^ T cells (Fig. 6B). As a result, the function of exhausted CD8^+^ T cells improved under Runx2 inhibition. Furthermore, we observed a decrease in PD-1 expression in cells treated with the Runx2 inhibitor compared with the control cells, suggesting that blocking Runx2 reduces the expression of exhaustion markers (Fig. 6C). These findings indicate that Runx2 inhibitors can potentially slow or inhibit the progression of T-cell exhaustion. Additionally, the Runx2 inhibitor decreased proliferation and enhanced apoptosis in Hepa1-6 cancer cells, suggesting that Runx2 inhibition not only improves CD8^+^ T-cell function but also suppresses growth in cancer cells, potentially enhancing the efficacy of immunotherapy (Fig. S14). We then evaluated the cytotoxic capacity of CD8^+^ T cells after treatment with either the Runx2 inhibitor or vehicle. We treated P14 CD8^+^ T cells to form suitable condition with either the Runx2 inhibitor or vehicle, then coculturing exhausted P14 CD8^+^ T cells with Hepa1‒6 gp33 cancer cells for 30 h (Fig. 6D). Given that P14 CD8^+^ T cells specifically recognize the gp33 peptide, they effectively target Hepa1‒6 gp33 cancer cells. Compared with vehicle treatment, treatment with the Runx2 inhibitor significantly increased the cytotoxic capacity, further supporting the role of Runx2 in modulating T-cell exhaustion and its potential as a target for improving immunotherapy responses (Fig. 6E). To further explore mechanisms of resistance, we established an anti-PD1-resistant hepatocellular carcinoma (HCC) cell line. Hepa1-6 cells were subcutaneously implanted into the right flank of C57BL/6 mice, followed by anti-PD1 antibody treatment. As expected, tumors initially regressed upon treatment; however, they eventually developed resistance, characterized by rapid regrowth and loss of responsiveness to anti-PD1 therapy. Tumors exhibiting acquired resistance were harvested, and cancer cells were sorted and cultured to generate a resistant primary cell line (Fig. 6F). To confirm the resistant phenotype, we re-implanted the derived cell line into naïve C57BL/6 mice and observed that these tumors did not respond to anti-PD1 treatment, validating their resistance (Fig. 6G). Next, we evaluated whether targeting RUNX2 could overcome resistance using the RUNX2 inhibitor, CADD522, in this anti-PD1-resistant model. We hypothesized that RUNX2 plays a critical role in modulating treatment response and that its inhibition could improve therapeutic efficacy. Indeed, administration of CADD522 resulted in reduced tumor growth and significantly prolonged survival compared to vehicle-treated controls (Fig. 6H-I). These findings further supported the role of RUNX2 in regulating T-cell exhaustion and underscored its potential as a therapeutic target for overcoming immune checkpoint inhibitor resistance.

## Discussion

The fundamental factors driving T-cell exhaustion and unfavorable clinical outcomes after ICI therapy remain poorly understood. Here, we identified *Runx2* as a crucial TF in the process of T-cell exhaustion after anti-PD1 treatment through single-cell multiome analysis of both an in vivo model and an ex vivo model. Runx2, a TF belonging to the RUNX family, is widely recognized as essential for osteoblast differentiation [33, 34]. Other members of the RUNX family, such as Runx1 and Runx3, have been implicated in promoting T-cell differentiation, indicating that the RUNX family may play crucial roles in determining the differentiation path of T cells [35, 36]. Although the role of Runx2 in T lymphocytes remains unclear, some studies have shown that Runx2 is enriched in exhausted CD8^+^ T cells, which suggests that Runx2 might be a crucial regulator in the process of T-cell exhaustion after ICI therapy [37, 38].

The regulatory mechanisms underlying progressive T-cell exhaustion in HCC contribute significantly to the failure of ICI therapy, which plays pivotal roles in dampening T-cell activation and effector function, highlighting the importance of understanding and targeting these regulatory pathways to overcome acquired resistance in HCC [39, 40]. In this study, we identified Runx2 not only as a major regulatory TF of T-cell exhaustion in HCC but also as a downstream target gene of Runx2, reinforcing its roles in the regulation of progressive T-cell exhaustion. Notably, we identified eight Runx2 targets, including Ctla4, Stat3, Klrk1, and Lgals3, as T-cell exhaustion-regulating genes. Accordingly, CTLA-4, a well-known immune checkpoint receptor found exclusively on T cells, may play a role in preventing T-cell reactivation, leading to acquired resistance. In addition, STAT3 has been demonstrated to induce PD-1 expression and suppress effector T cells, which contributes to T-cell exhaustion [41]. Furthermore, STAT3 is known as a downstream effector and key mediator of the JAK/STAT3 signaling pathway, which suppresses antitumor immunity [42]. Similarly, through pathway enrichment analysis, we revealed that the JAK/STAT signaling pathway was highly activated in exhausted CD8^+^ T cells (Fig. 3B). Moreover, KLRK1, a member of the KLR family, has been treated as a marker of exhausted CD8^+^ T cells. These findings suggest that the regulation of Runx2 in T cells affects the progress of T-cell exhaustion upon ICI treatment in HCC. In light of the findings presented in this study, the relationships and mechanistic activities of several genes targeted by Runx2 remain unclear, representing a significant avenue for future research.

We collected tumor-infiltrating lymphocytes to mimic patients via an in vivo HCC mouse model. In addition, to explore the associated mechanism and establish an accessible source of exhausted CD8^+^ T cells, we developed an ex vivo P14 CD8^+^ T-cell model. We collected splenocytes from P14 mice and induced their exhaustion through repeated gp33 stimulation. Because P14 CD8^+^ T cells can recognize the gp33 peptide specifically, we exposed CD8^+^ T cells to this peptide to mimic conditions such as chronic infection or the tumor microenvironment to induce exhaustion [43–45]. Exhausted CD8^+^ T cells express high levels of inhibitory receptors (such as PD-1 and LAG3) and low levels of effector cytokines (such as TNF-α, IFN-γ, IL-2, and Granzyme B), expressing increased levels of transcription factors related to exhaustion (such as TOX and EOMES) and decreased cytotoxic capacity [46–49]. To identify exhausted CD8^+^ T cells in this ex vivo model, we detected inhibitory receptors, effector cytokines, and transcription factors and compared them to those of naïve and active CD8^+^ T cells. These data showed that exhaustion had been induced and that these exhausted CD8^+^ T cells indeed had a reduced capacity to kill cancer cells compared with active CD8^+^ T cells (online supplemental Fig. 6). This concept of an ex vivo CD8^+^ T-cell induced exhaustion model has also been utilized by other research groups to investigate associated mechanisms, yielding similar results [50]. Their studies demonstrated the ex vivo model effectively replicates key features of exhausted CD8^+^ T cells, establishing it as a viable alternative to the in vivo LCMV model [50]. Consequently, the ex vivo CD8^+^ T-cell-induced exhaustion model serves as a valuable tool for exploring the detailed molecular mechanisms involved in gene expression regulation.

## Conclusion

The results of our study identified Runx2 as a key regulator of the progressive CD8^+^ T-cell exhaustion induced by ICIs in HCC. We identified the downstream targets regulated by Runx2 and delineated the path of CD8^+^ T-cell exhaustion following prolonged ICI treatment. Crucially, our findings establish a link between the regulatory network governing CD8^+^ T-cell exhaustion in murine models and the clinical response observed in HCC patients receiving ICI therapy. The discovery of Runx2 regulation and its target genes offers a promising avenue for preventing T cells from entering a progressively exhausted state and presents a potential therapeutic option for combination with immunotherapy to overcome acquired resistance.

## Supplementary Information

Supplementary material 1

## Data Availability

All data needed to evaluate the conclusions in the paper are present in the paper and/or the Supplemental Materials. The raw and processed data included in this study have been deposited in NCBI’s Gene Expression Omnibus (GEO) archive (https://www.ncbi.nlm.nih.gov/geo) and are accessible through GEO SuperSeries accession number GSE268165; in vivo single-cell RNA-seq data are available through GEO accession number GSE268163 and ex vivo single-cell ATAC-seq and RNA-seq data are available under GEO accession numbers GSE268160 and GSE268161, respectively. Moreover, all the codes used in this study, including R scripts and Python source codes, are available via our GitHub page (https://github.com/yangtseng/T_cell_exhaustion/).

## Abbreviations

AUC: Area under the curve
CTLA-4: Cytotoxic T-lymphocyte-associated antigen 4
GO: Gene ontology
GRN: Gene regulatory network
HCC: Hepatocellular carcinoma
HVG: Highly variable genef
ICI: Immune checkpoint inhibitor
KEGG: Kyoto encyclopedia of genes and genomes
LSI: Latent semantic indexing
MAD: Median absolute deviation
MSigDB: Molecular signature database
ORA: Over-representation analysis
PC: Principal component
PCA: Principal component analysis
PD-1: Programmed death-1
PD-L1: Programmed death-ligand 1
PI: Propidium iodide
scATAC-seq: Single-cell assay for transposase-accessible chromatin with sequencing
scRNA-seq: Single-cell RNA sequencing
TF: Transcription factor
TF-IDF: Term frequency-inverse document frequency
SVD: Singular value decomposition
UMAP: Unified manifold approximation and projection
