# Supplementary material for: Transcriptional dynamics of CD8+ T-cell exhaustion in immune checkpoint inhibitor resistance at single-cell resolution
Source: Mol Cancer. 2025 Dec 11;24:306. doi: 10.1186/s12943-025-02468-7 (PMC12696930; doi:10.1186/s12943-025-02468-7)
Supplement: Supplementary file 1 — Supplementary material 1 [file 12943_2025_2468_MOESM1_ESM.docx]

**Supplemental material**

**Fig. S1. Characteristics of *in vivo* model CD8^+^ T cells.** (A) UMAP plot of the cell composition of entire tumor samples. (B) The expression level of the well-known T-cell marker *Cd3e*. (C) The expression level of the well-known CD8^+^ T-cell marker *Cd8a*.

**Fig. S2. Characteristics of CD8^+^ T cells from the *in vivo* model.** (A) UMAP plot of the CD8^+^ T-cell composition of each sample. (B) Stacked bar plot showing the proportion of cells in each group (left). The bar plot shows the composition of the number of cells in each group (right).

**Fig. S3. CD8^+^ T cells subtypes from the *in vivo* model.** (A) Cells are colored according to the expression level of well-known T-cell subtype marker genes, including effector T cells (Cd7, Gzmb and Gzmk), exhausted T cells (Ctla4, Lag3 and Pdcd1), high-IFN response T cells (Ifit1, Ifit3 and Isg15), memory T cells (Il7r, Tcf7 and S1pr1) and proliferative T cells (Ccna2, Ccnb2 and Cdk1). (B) Dot plots revealed the expression level of each marker in 9 clusters. (C) Results of CD8+ T-cell type annotation. IFN, interferon. (D) Stacked bar plot showing the proportion of cells in each T-cell subtype (left). The bar plot shows the composition of the number of cells in each T-cell subtype (right).

**Fig. S4. Analysis of RNA velocity in exhausted CD8^+^ T cells from an *in vivo* model.** (A) Proportion of spliced and unspliced RNA in the entire T-cell population (upper panel). Proportions of spliced and unspliced RNA in each T-cell cluster (lower panel). (B) Velocity embedding of the entire CD8^+^ T-cell population from the mouse model. (C) The color on the UMAP represents the latent time of each cell determined through dynamic modeling, indicating exhausted T cells (cluster 1) and memory T cells (cluster 3) as two poles of the CD8^+^ T-cell population.


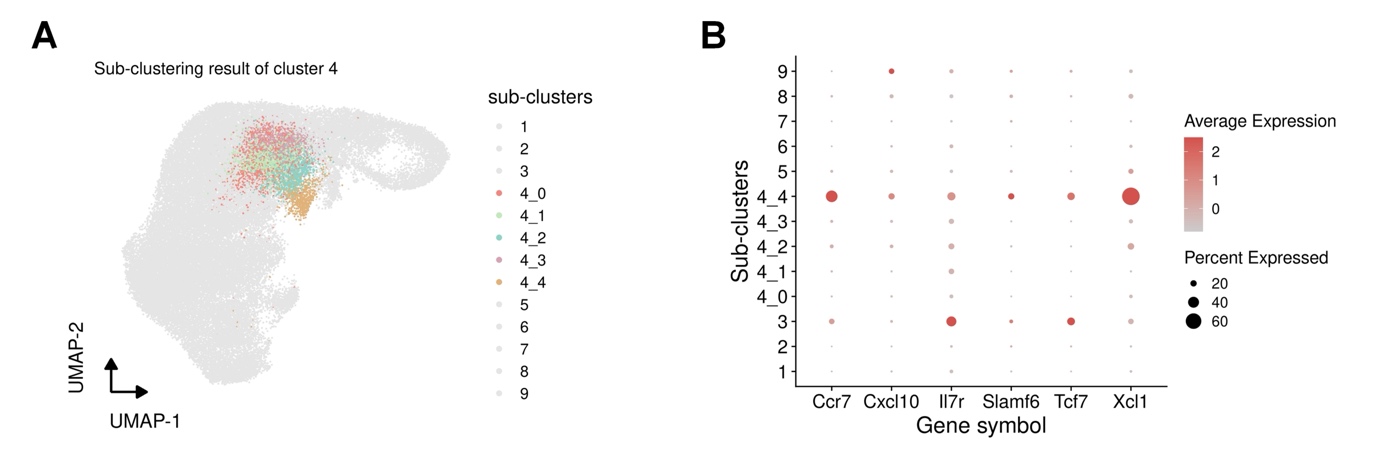


**Fig. S5. Subclustering revealed a pure progenitor population of exhausted CD8^+^ T cells.** (A) We performed subclustering of cluster 4, which expressed high levels of well-known exhausted CD8^+^ T-cell progenitor markers, to identify a pure exhausted CD8^+^ T-cell progenitor population. (B) Dot plot showing the expression patterns of selected progenitor markers that were highly expressed in subcluster 4_4.

**Fig. S6. Characteristics of exhausted CD8^+^ T cells from the *ex vivo* model.** (A-C) Identification of induced CD8^+^ T-cell statuses *ex vivo*. We assessed exhaustion markers and associated transcription factors in naïve, active, and exhausted CD8^+^ T cells by flow cytometry. (D) Cytotoxic capacity of different CD8^+^ T-cell statuses. We cocultured 5x10^5 target cells with varying numbers of naïve, active, or exhausted CD8^+^ T cells as effectors. After 24 hours of coculture, we harvested the cells, stained them with live/dead dye and the marker CD45, and analyzed them by flow cytometry. Asterisks indicate the relative cytotoxic capacities of active versus exhausted CD8^+^ T cells. *p<0.05; **p<0.01; ***p<0.001; ****p<0.0001, two-tailed Student’s t test. Data are presented as mean ± standard error (SE).


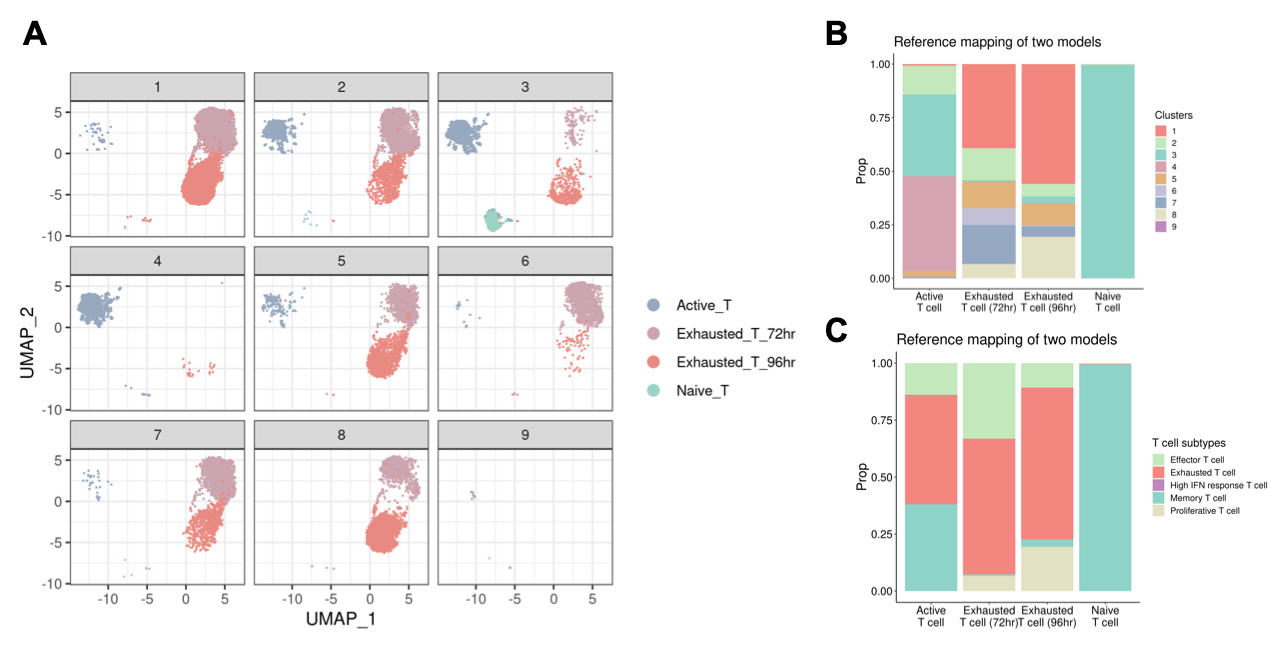


**Fig. S7. Reference mapping between two models.** (A) UMAP visualization showed the mapping result of *ex vivo* model onto each cluster from *in vivo* model. (B-C) Stacked bar plot revealed the composition of each *ex vivo* group within *in vivo* clusters (B) and *in vivo* subtypes (C).

**
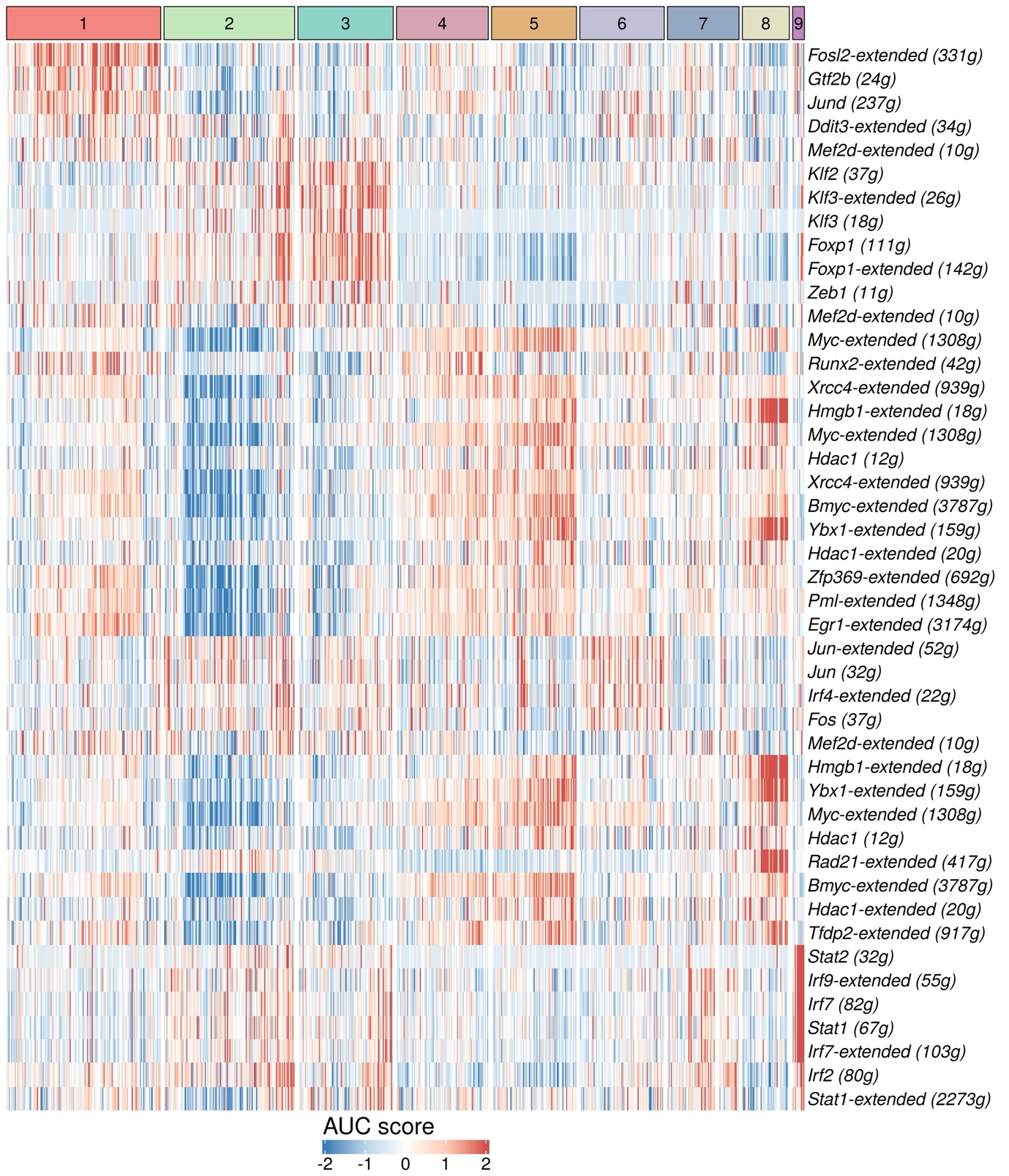
**

**Fig. S8. Potential transcription factors in T cells from *in vivo* model.** Significantly highly expressed TFs within each cluster were selected (p value < 0.05) and presented via a heatmap, with the color representing the AUC score.

**
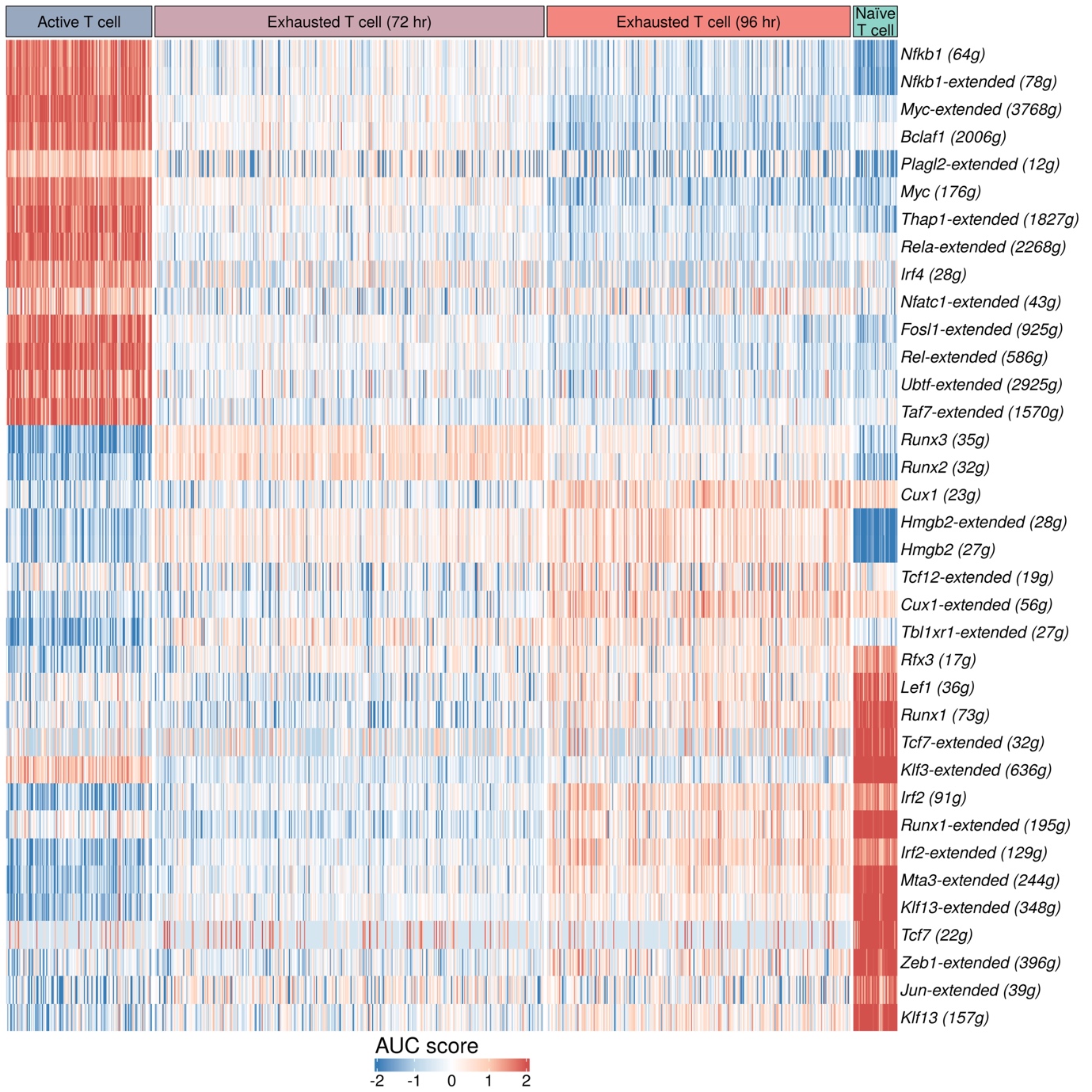
**

**Fig. S9. Potential transcription factors in T cells from *ex vivo* model.** Significantly highly expressed TFs within each cluster were selected (p value < 0.05) and presented via a heatmap, with the color representing the AUC score.

**Fig. S10. Over-representation analysis revealed significant overlap between Runx2 regulome and T-cell activation.** (A) Significantly overlapped pathways from GO biological process, with Runx2 regulome, were selected (p value < 0.01 and Gene Ratio > 0.1) and presented via a dotplot. (B) Significantly overlapped pathways from KEGG, with Runx2 regulome, were selected (p value < 0.01 and Gene Ratio > 0.1) and presented via a dotplot.

**Fig. S11. Relationship between the *Runx2* signature and T-cell exhaustion signature.** (A) Dot plot illustrating the gene expression patterns of Runx2 and its downstream targets in each T-cell cluster. (B) UMAP plot of the *Runx2* signature level on a two-dimensional plane. (C) Violin plot indicating the level of *Runx2* signature expression in each T-cell subtype. (D) Violin plot indicating the level of *Runx2* signature expression in each T-cell cluster, which was elevated in clusters 1 and 4. (E) Pearson correlations between the *Runx2* signature and the T-cell exhaustion signature in different T-cell subtypes.


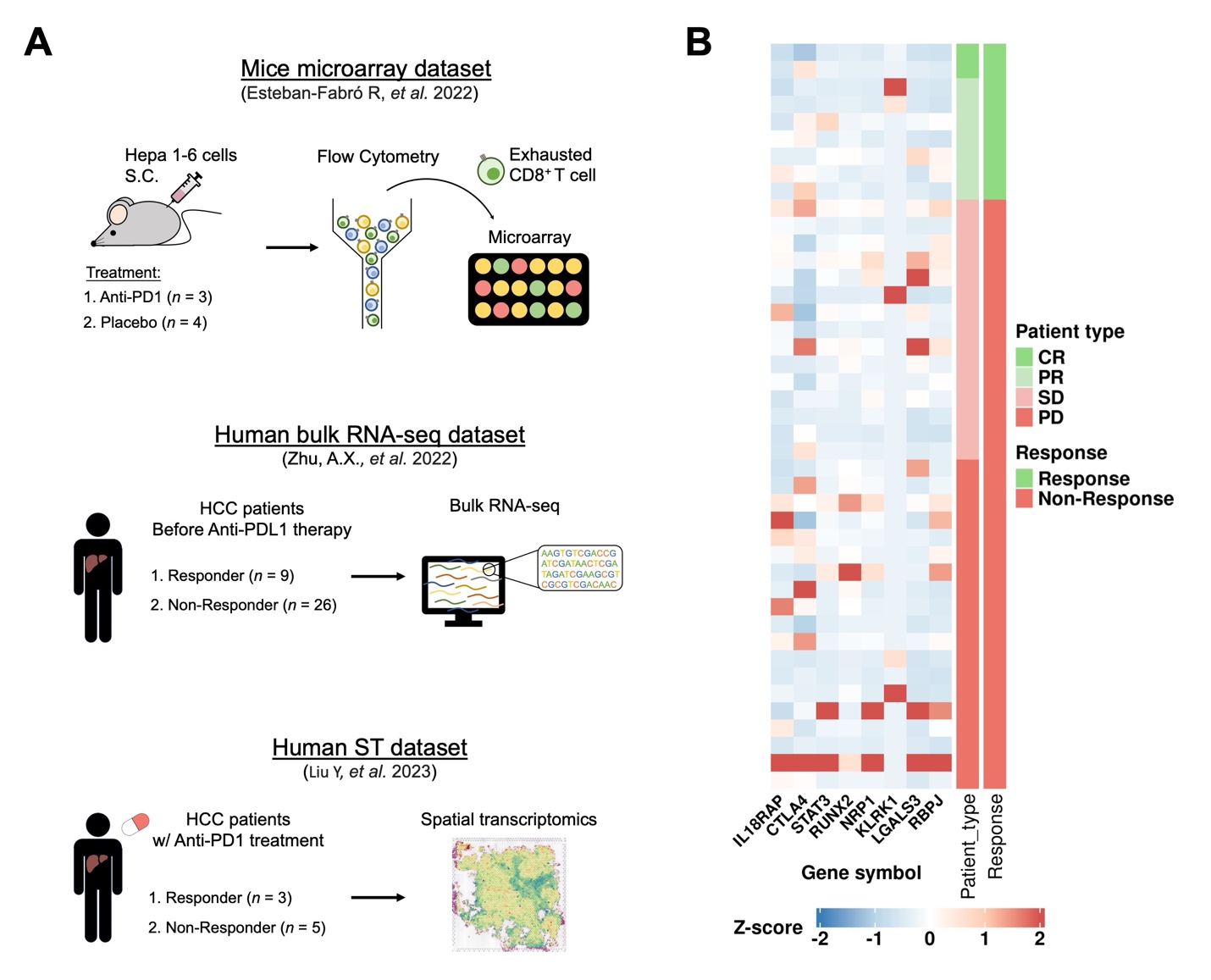


**Fig. S12. External validation of the *Runx2* regulon in three datasets.** (A) Scheme shows the experimental design of three external datasets. (B) Heatmap indicates that patients who did not respond to anti-PDL1 therapy had increased expression levels of *RUNX2* and its downstream targets.


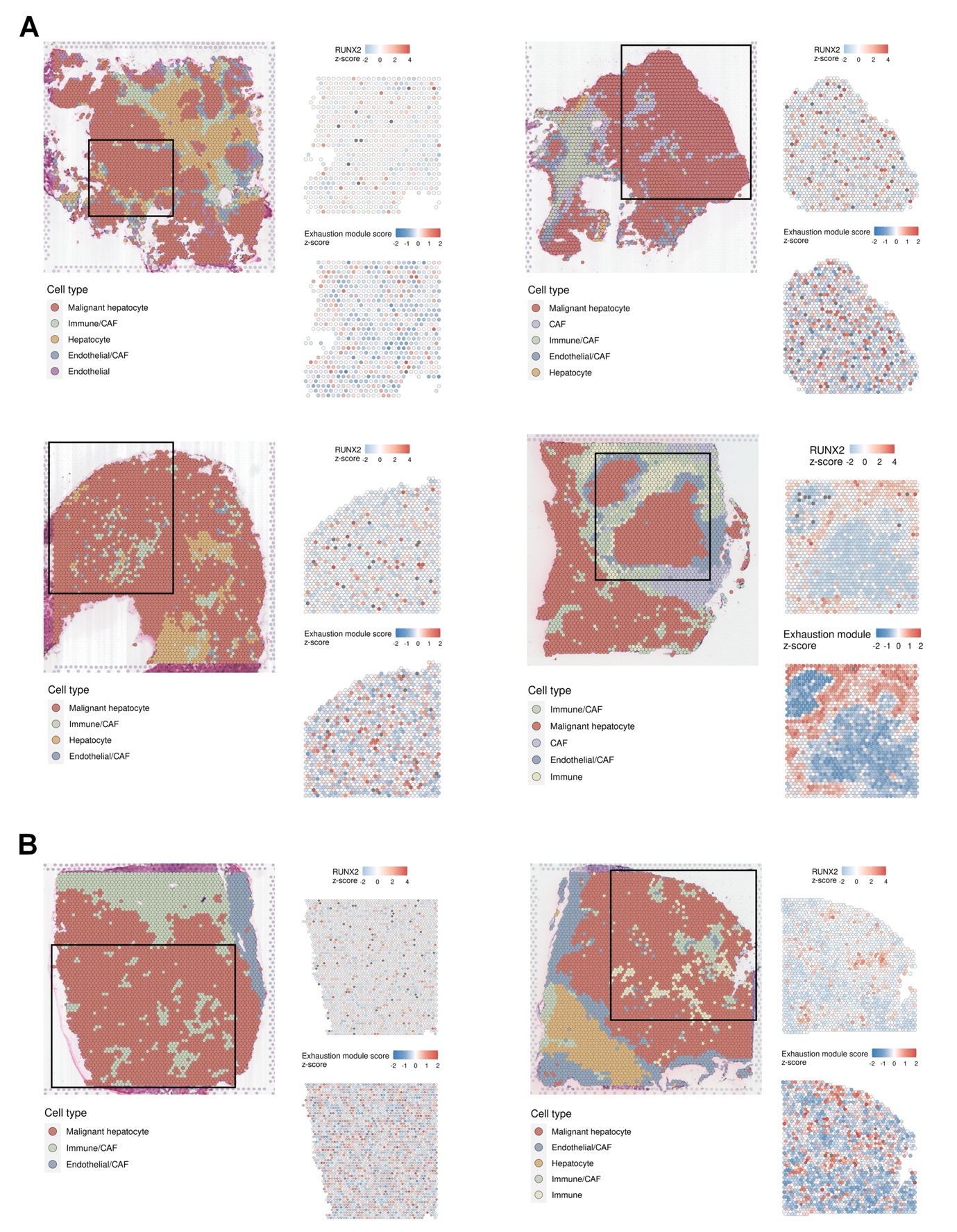


**Fig. S13. External validation of the role of *Runx2* in the ICI response using a human spatial transcriptomics dataset.** (A) Spatial transcriptomics datasets from four ICI-nonresponsive patients were reannotated (left panel). We also evaluated the *Runx2* expression level and exhaustion module score of the carcinoma region (right panel), which were strongly correlated with the *Runx2* expression level and exhaustion module score in immune cells. (B) Spatial transcriptomics datasets from two ICI-responsive patients were reannotated (left panel). We also evaluated the *Runx2* expression level and exhaustion module score of the carcinoma region (right panel), which revealed a weak correlation between the *Runx2* expression level and the exhaustion module score in immune cells.

**Fig. S14. The Runx2 inhibitor induced death in Hepa1‒6 liver cancer cells.** (A) We treated Hepa1-6 liver cancer cells with various concentrations of the Runx2 inhibitor CADD522 for 96 hours and assessed cell viability via MTT assay. (B) After treatment with either 0 or 100 µM Runx2 inhibitor for 96 hours, we harvested the Hepa1-6 cancer cells and analyzed annexin V expression via flow cytometry. ***p<0.001, two-tailed Student’s t test. Data are presented as mean ± standard error (SE).

**Supplemental Table 1. Cell markers for spatial transcriptomics dataset reannotation.**

| **Cell types** | **Gene** | **Gene symbol** |
| --- | --- | --- |
| T cells | Interleukin 7 receptor | *IL7R* |
|  | CD3 antigen, delta polypeptide | *CD3D* |
|  | CD8 Subunit Alpha | *CD8A* |
| Dendritic cells | Lysozyme | *LYZ* |
|  | CD68 molecule | *CD68* |
|  | Complement C1q B Chain | *C1QB* |
| B cells | B cell scafoold protein | *BANK1* |
|  | CD19 molecule | *CD19* |
|  | CD22 molecule | *CD22* |
| Fibroblasts | Collagen type I alpha 2 chain | *COL1A2* |
|  | Familial adenomatous polyposis | *FAP* |
|  | Decorin | *DCN* |
| Endothelial cells | Platelet and endothelial cell adhesion molecule 1 | *PECAM1* |
|  | Cadherin 5 | *CDH5* |
|  | Stanniocalcin-1 | *STC1* |
| Carcinoma | Alpha-1-microglobulin/bikunin precursor | *AMBP* |
|  | Apolipoprotein | *APOH* |
|  | Prealbumin | *TTR* |
| Hepatocyte | Hepcidin antimicrobial peptide | *HAMP* |
|  | Cytochrome P450 1A2 | *CYP1A2* |
|  | Alcohol dehydrogenase 4 | *ADH4* |
| Monocytes | Transcription factor 4 | *TCF4* |
|  | CD80 molecule | *CD80* |
|  | CD86 molecule | *CD86* |

**Supplemental Table 2. Genes undergo feature enhancement through BayesSpace.**

| **Gene set** | **Gene** | **Gene symbol** |
| --- | --- | --- |
| Runx2 module | Runt related transcription factor 2 | *Runx2* |
|  | Cytotoxic T-lymphocyte associated protein 4 | *Ctla4* |
|  | Recombination signal binding protein for immunoglobulin kappa J region | *Rbpj* |
|  | Interleukin 18 receptor accessory protein | *Il18rap* |
|  | Lectin, galactose binding, soluble 3 | *Lgals3* |
|  | Killer cell lectin like receptor K1 | *Klrk1* |
|  | Neuropilin 1 | *Nrp1* |
|  | Signal transducer and activator of transcription 3 | *Stat3* |
| T cell | CD3 delta subunit of T-cell receptor complex | *Cd3d* |
|  | CD3 epsilon subunit of T-cell receptor complex | *Cd3e* |
|  | CD8 subunit alpha | *Cd8* |
| T cell exhaustion | Hepatitis A virus cellular receptor 2 | *Havcr2* |
|  | Lymphocyte-activation gene 3 | *Lag3* |
|  | Programmed cell death 1 | *Pdcd1* |
|  | T cell immunoreceptor with Ig and ITIM domains | *Tigit* |
|  | Thymocyte selection-associated high mobility group box | *Tox* |

**Supplemental Table 3. Cell counts of each sample from murine model.**

| **Sample name** | **Treatment** | **Time** | **Gene counts** | **Cell counts** |
| --- | --- | --- | --- | --- |
| P138A1 | Isotype | 20 days | 32,285 | 5,642 |
| P138A2 | Isotype | 20 days | 32,285 | 5,641 |
| P138B1 | Anti-PD-1 | 20 days | 32,285 | 9,428 |
| P138B2 | Anti-PD-1 | 20 days | 32,285 | 6,916 |
| P136A1 | Isotype | 57 days | 32,285 | 13,157 |
| P136A2 | Isotype | 57 days | 32,285 | 11,212 |
| P136B1 | Anti-PD-1 | 57 days | 32,285 | 14,349 |
| P136B2 | Anti-PD-1 | 57 days | 32,285 | 15,818 |

**Supplemental Table 4. Gene list of T cell function modules.**

| **Gene set** | **Gene** | **Gene symbol** |
| --- | --- | --- |
| T cell checkpoint | Cytotoxic T-lymphocyte associated protein 4 | *Ctla4* |
|  | Ectonucleoside triphosphate diphoshohydrolase 1 | *Entpd1* |
|  | Hepatitis A virus cellular receptor 2 | *Havcr2* |
|  | Lymphocyte-activation gene 3 | *Lag3* |
|  | Programmed cell death 1 | *Pdcd1* |
|  | T cell immunoreceptor with Ig and ITIM domains | *Tigit* |
| T cell effectory | Chemokine (C-C motif) ligand 3 | *Ccl3* |
|  | Chemokine (C-C motif) ligand 4 | *Ccl4* |
|  | Granzyme B | *Gzmb* |
|  | Granzyme K | *Gzmk* |
|  | Interferon γ | *Ifng* |
|  | Natural killer cell group 7 sequence | *Nkg7* |
|  | Perforin 1 | *Prf1* |
| T cell exhaustion | Cd244 molecule A | *Cd244a* |
|  | Eomesodermin | *Eomes* |
|  | Lymphocyte-activation gene 3 | *Lag3* |
|  | Programmed cell death 1 | *Pdcd1* |
|  | T cell immunoreceptor with Ig and ITIM domains | *Tigit* |
|  | Thymocyte selection-associated high mobility group box | *Tox* |

**Supplemental Table 5. Direct inhibitors of Runx2 and its downstream target genes.**

| **Targets** | **Direct inhibitors** |
| --- | --- |
| *Runx2* | CADD522 |
| *Lgals3* | GB1107 |
| *Nrp1* | EG00229 |
| *Stat3* | WP1066 |
